# Novel genetic determinants contribute to hearing loss in a central European cohort with enlarged vestibular aqueduct

**DOI:** 10.1186/s10020-025-01159-9

**Published:** 2025-03-22

**Authors:** Emanuele Bernardinelli, Raffaella Liuni, Rapolas Jamontas, Paola Tesolin, Anna Morgan, Giorgia Girotto, Sebastian Roesch, Silvia Dossena

**Affiliations:** 1 Institute of Pharmacology and Toxicology, Paracelsus Medical University, Strubergasse 21, 5020 Salzburg, Austria; 2 Medical Genetics, Institute for Maternal and Child Health-IRCCS Burlo Garofolo, 34137 Trieste, Italy; 3 Department of Medicine, Surgery and Health Sciences, University of Trieste, 34137 Trieste, Italy; 4 Department of Otorhinolaryngology, Head and Neck Surgery, Paracelsus Medical University, 5020 Salzburg, Austria; 5 Department of Otorhinolaryngology, University Hospital Regensburg, 93053 Regensburg, Germany; 6 Research and Innovation Center Regenerative Medicine and Novel Therapies (FIZ RM&NT), Paracelsus Medical University, 5020 Salzburg, Austria; 7 Present Address: Department of Molecular Microbiology and Biotechnology, Institute of Biochemistry, Life Sciences Center, Vilnius University, 10257 Vilnius, Lithuania

**Keywords:** Enlarged vestibular aqueduct, Hearing loss, Pathogenic variants, *SLC26A4*, *TJP2*

## Abstract

**Background:**

The enlarged vestibular aqueduct (EVA) is the most commonly detected inner ear malformation. Biallelic pathogenic variants in the *SLC26A4* gene, coding for the anion exchanger pendrin, are frequently involved in determining Pendred syndrome and nonsyndromic autosomal recessive hearing loss DFNB4 in EVA patients. In Caucasian cohorts, the genetic determinants of EVA remain unknown in approximately 50% of cases. We have recruited a cohort of 32 Austrian patients with hearing loss and EVA to define the prevalence and type of pathogenic sequence alterations in *SLC26A4* and discover novel EVA-associated genes.

**Methods:**

Sanger sequencing, single nucleotide polymorphism (SNP) assays, copy number variation (CNV) testing, and Exome Sequencing (ES) were employed for gene analysis. Cell-based functional and molecular assays were used to discriminate between gene variants with and without impact on protein function.

**Results:**

*SLC26A4* biallelic variants were detected in 5/32 patients (16%) and monoallelic variants in 5/32 patients (16%). The pathogenicity of the uncharacterized SLC26A4 protein variants was assigned or excluded based on their ion transport function and cellular abundance. The monoallelic or biallelic Caucasian EVA haplotype was detected in 7/32 (22%) patients, but its pathogenicity could not be confirmed. X-linked pathogenic variants in *POU3F4* (2/32, 6%) and biallelic pathogenic variants in *GJB2* (2/32, 6%) were also found. No CNV of *SLC26A4* and *STRC* genes was detected. ES of eleven undiagnosed patients with bilateral EVA detected rare sequence variants in six EVA-unrelated genes (monoallelic variants in *SCD5, REST, EDNRB, TJP2, TMC1*, and two variants in *CDH23*) in five patients (5/11, 45%). Cell-based assays showed that the *TJP2* variant leads to a mislocalized protein product forming dimers with the wild-type, supporting autosomal dominant pathogenicity. The genetic causes of hearing loss and EVA remained unidentified in (14/32) 44% of patients.

**Conclusions:**

The present investigation confirms the role of *SLC26A4* in determining hearing loss with EVA, identifies novel genes in this pathophysiological context, highlights the importance of functional testing to exclude or assign pathogenicity of a given gene variant, proposes a possible diagnostic workflow, suggests a novel pathomechanism of disease for *TJP2*, and highlights voids of knowledge that deserve further investigation.

**Supplementary Information:**

The online version contains supplementary material available at 10.1186/s10020-025-01159-9.

## Background

Hearing loss affects 1.5 in 1000 newborns, is the most common sensory deficit in humans, and is a significant cause of disability in children (Koffler et al. 2015; Choe et al. 2023). Genetics accounts for at least 60% of cases of hearing loss in developed countries (Pandya 2016) and more than 150 genes are known to be involved in the function of hearing, making the genetic diagnostics of hearing loss a challenging issue (Hereditary Hearing Loss Homepage 2024). Identifying the gene causative for hearing loss provides a conclusive diagnosis to patients and their families and is fundamental for a reliable prognosis, genetic counseling, and planning of an adequate intervention (Jonard et al. 2023). Hearing loss of genetic origin is frequently associated with inner ear malformations (Sennaroglu and Saatci 2002), of which the most common is the enlarged vestibular aqueduct (EVA) (Usami et al. 1999). EVA is inherited in an autosomal recessive manner, is often associated with cochlear incomplete partitions, and can be found in both syndromic and non-syndromic forms of hearing loss. Genes that have been implied in the pathogenesis of non-syndromic EVA are *SLC26A4, GJB2, FOXI1, KCNJ10,* and *POU3F4* (Roesch et al. 2021).

Pathogenic sequence alterations of the *SLC26A4* gene, which encodes for the anion exchanger pendrin (OMIM *605646), lead to EVA in the context of autosomal recessive Pendred syndrome (OMIM #274600) or non-syndromic deafness DFNB4 (OMIM #600791) (Everett et al. 1997). In DFNB4, sensorineural hearing loss with or without vestibular dysfunction is the only clinical feature, while in Pendred syndrome the hearing loss is associated with a partial iodide organification defect that usually appears around puberty and may lead to subclinical or overt hypothyroidism with or without goiter (Fugazzola et al. 2001). Hearing loss in DFNB4/Pendred syndrome typically has an early onset in childhood and is moderate to severe and stable, but can also be fluctuating and progressive and appear later in life after a head trauma or barotrauma (Smith et al. 1993; Griffith and Wangemann 2011).

While Pendred syndrome is invariably linked to biallelic pathogenic sequence alterations in the *SLC26A4* gene, in Caucasian cohorts these are found only in approximately 25% of EVA patients. Of the remaining EVA patients, 25% harbor monoallelic *SLC26A4* sequence alterations with the second mutated allele that remains unidentified, and 50% are negative for *SLC26A4* (Ito et al. 2013). Additional genes that have been linked to EVA are *FOXI1* and *KCNJ10*. *FOXI1* (OMIM *601093) codes for a transcription factor of *SLC26A4* (Hulander et al. 2003; Yang et al. 2007), and *KCNJ10* (OMIM *602208) encodes the inwardly rectifying potassium channel Kir4.1, which is essential for the maintenance of the endocochlear potential (Marcus et al. 2002). Digenic inheritance of EVA caused by one sequence alteration in *SLC26A4* and another in *FOXI1* or *KCNJ10* has been suggested (Yang et al. 2007; Yang et al. 2009), but this genetic configuration is infrequent in Caucasian cohorts (Landa et al. 2013; Pique et al. 2014; Cirello et al. 2012), and its significance is uncertain. In a minority (0–8%) of EVA patients, pathogenic sequence alterations in *GJB2* (OMIM *121011) are detected (Kenna et al. 2011; Lee et al. 2009; Propst et al. 2006). However, whether these findings are causative or coincidental is not unequivocally established, and EVA in these patients is likely due to factors other than *GJB2* (Roesch et al. 2021). Mutations in *POU3F4* (OMIM *300039), which lead to X-linked DFN3/DFNX2 (OMIM #304400) associated with cochlear incomplete partition type 3 (IP3), are rare in EVA cohorts, and from 0 to 50% of *POU3F4* patients have been found to have an EVA (de Kok et al. 1995; Pollak et al. 2016; Gong et al. 2014).

Recently, a new haplotype called Caucasian EVA (CEVA) was reported in several patients with monoallelic pathogenic sequence alterations in *SLC26A4* and in some patients with no pathogenic variants in known causative genes (Chattaraj et al. 2017). The CEVA haplotype consists of 12 single nucleotide polymorphisms (SNPs), including 10 single nucleotide substitutions and 2 single nucleotide deletions, falling in intergenic regions or non-coding genomic regions of genes far upstream *SLC26A4* and correlates with phenotype severity in EVA patients (Chao et al. 2019).

Overall, *SLC26A4* and the CEVA haplotype, *GJB2*, *FOXI1*, *KCNJ10,* and *POU3F4* may account for hearing loss and EVA in little or no more than 50% of patients in Caucasian cohorts, and the causative gene remains unidentified in 50% of patients. We formerly reported that *SLC26A4* pathogenic variants are underrepresented in our Austrian cohort of patients with hearing loss and EVA compared to other Caucasian cohorts (Roesch et al. 2018). Thus, the analysis of this cohort might reveal novel genetic factors linked to EVA. Here, we combine molecular genetics and functional tests to identify the causative gene in our expanded cohort. Exome sequencing (ES) in patients negative for the known causative genes allowed for the identification of novel genes formerly unrelated to EVA. Cell-based assays permitted discrimination between gene variants with no impact on protein function and pathogenic sequence alterations, linking a given genotype with the clinical phenotype.

## Material and methods

### Patient recruitment

Patients have been recruited among those referred to the Otolaryngology department of the Salzburg General Hospital for hearing loss. A complete audiological examination, family history of hearing loss, and informed consent for gene analysis for diagnostics and research were obtained from all patients or their legal representatives. The research was prospectively reviewed and approved by a duly constituted ethics committee (approval 415-E/2092/6-2017 for gene analysis and 415-E/2548/13-2019 for circulating nucleic acid analysis) and has therefore been performed in accordance with the principles embodied in the 1964 Declaration of Helsinki and its later amendments (Available online: https://www.wma.net/policies-post/wma-declaration-of-helsinki-ethical-principles-for-medical-research-involving-human-subjects/).

Thirty-two Austrian subjects (16 females and 16 males aged between 5 and 65 years; median age 37 years, average age 34 years) with hearing loss and EVA were included in the study. Imaging studies of the inner ear by computer tomography (CT) of the temporal bones were performed. EVA was defined according to the Cincinnati criteria [vestibular aqueduct at midpoint and operculum > 0.9 and > 1.9 mm, respectively (Vijayasekaran et al. 2007)]. An abnormal cochlea was considered an incomplete partition type 2 (IP2) in cases of a normal basal turn and cavity-like appearing distal turns with a missing interscalar ridge between the basal turn and the distal turns on the axial plane of the CT scan (Leung et al. 2016). Parameters for individual characterization of hearing loss (HL) are given in the Additional files 1 and 2.

Magnetic resonance imaging (MRI) of the cerebellopontine angle (variable manufacturers) was analyzed by assessment of T2-weightend, axial planes of both sides.

Vestibular testing was performed with the Interacoustics VisualEyes™ 515 System, including the EyeSeeCam for video head impulse testing and caloric testing with water or air irrigation, depending on the individual presence of a tympanic membrane perforation.

The thyroid function was evaluated based on the presence of overt goiter, the results of the perchlorate discharge test in adults, thyroid ultrasound, or alterations of laboratory functional parameters in selected patients.

### Gene analysis

Patient whole blood was collected in plastic tubes with potassium-ethylenediaminetetraacetic acid (S-Monovette^®^, Sarstedt, Nümbrecht, Germany) via venipuncture. Total genomic DNA (gDNA) was purified from ~ 350 μL blood with the EZ1 DSP DNA Blood 350 μL kit (Qiagen, Hilden, Germany) using the EZ1 Advanced XL platform (Qiagen) according to the manufacturer’s instructions. Quantification was performed with the QIAxpert (Qiagen) spectrophotometer. Only samples with an A260/A280 between 1.7 and 1.9 were used for downstream analysis.

The 21 exons and intron–exon boundaries of *SLC26A4* (NCBI GeneBank Ref. Sequence: NG_008489.1) and the coding sequence of *FOXI1* (NG_012068.1), *GJB2* (NG_008358.1), *GJB3* (NG_008309.1), *POU3F4* (NG_009936.2), and *KCNJ10* (NG_016411.1) have been amplified by endpoint PCR from gDNA samples. Fifty μL endpoint polymerase chain reaction (PCR) reactions contained 1 × JumpStart REDAccuTaq Long and Accurate (JS RAT LA) DNA Polymerase buffer (Sigma; St. Louis, MO, USA), 20 mM dNTPs (Thermo Fisher Scientific; Waltham, MA, USA), 20% dimethyl sulfoxide (Sigma), 0.4–0.8 μM forward and reverse primers and 2.5 units JS RAT LA DNA Polymerase (Sigma). *SLC26A4* and *GJB2* amplification and sequencing primers have already been described (Roesch et al. 2018). Amplification and sequencing primers for *FOXI1*, *GJB3*, *POU3F4*, and *KCNJ10* are described in the Additional file 2: Table S1. The PCR products were purified and Sanger sequenced (Microsynth AG, Balgach, Switzerland), and the resulting sequences were compared against the NCBI DNA reference sequence assembly with MacVector (version 18.6.4). In the case of the detection of an exonic sequence or splice site variant, the results have been confirmed on an independent amplicon to exclude errors of the DNA polymerase. The configuration (*cis* or *trans*) of biallelic variants was determined by analysis of parents of index patients.

The presence of the two common genomic deletions del(GJB6-D13S1830) and del(GJB6-D13S1854) have been verified by multiplex PCR as previously described (del Castillo et al. 2005).

The possible presence of the CEVA haplotype SNPs rs17424561, rs79579403, rs17425867, rs117113959, rs17349280, rs117386523, rs80149210, rs9649298, rs117714350, rs150942317, and rs199667576 has been determined by the rhAmp^®^ SNP Assays (Integrated DNA Technologies, Coralville, IA, USA) run on the Rotor Gene (Qiagene) instrument. The SNP rs199915614 has been verified by Sanger sequencing with the primers indicated in Additional file 2: Table S1.

The copy numbers of selected genes were determined with the QuantStudio^®^ 3D Digital PCR (Life Technologies, Thermo). A master mix including the gDNA, the QuantStudio™ 3D Digital PCR Master Mix (Applied Biosystems, Thermo, Waltham, MA, USA), the CNV Assay for the target gene (*SLC26A4*: Hs02774758; *STRC*: STRC_CDVMKD7) and the CNV Assay for a reference gene (*RNAseP*: 4401631; Telomerase reverse transcriptase, *TERT*: 4401633) was loaded on a QuantStudio™ 3D Digital PCR 20 K Chip in a semi-automated manner with a QuantStudio™ 3D Digital PCR Chip loader (Applied Biosystems) and submerged in the immersion fluid provided by the vendor. After sealing the chip, a PCR reaction was performed on ProFlex PCR System (Life Technologies). The fluorescence signal was read with the QuantStudio^®^ 3D and the results were uploaded to the online QuantStudio^®^ 3D Analysis Suite™ for evaluation. The quality of the analysis was evaluated and manually confirmed. The number of copies per µl calculated for the target gene was normalized for the number of copies per µl of the reference gene (*RNAseP* or *TERT*).

### Exome sequencing and variants filtering

As a first step, 50 ng of gDNA underwent enzymatic fragmentation, followed by end repair and dA-tailing reactions. Subsequently, the DNA fragments were ligated to a universal adapter and amplified using the Unique Dual Index primer. According to the manufacturer's instructions, genomic libraries were generated with the Twist Human Core Exome + Human RefSeq Panel kit (Twist Bioscience, South San Francisco, CA, USA). Finally, the hybridized fragments were captured and amplified, and ES was carried out on an Illumina NextSeq 550 instrument (Illumina Inc., San Diego, CA, USA).

This process initially generates FASTQ files, which are processed through a custom pipeline (Germline-Pipeline) developed by enGenome srl to create VCF files. Those files contain germline variants, such as Single Nucleotide Variants (SNVs) and short insertion/deletions (INDELs), and were analyzed with the enGenome Expert Variant Interpreter (eVai) software (evai.engenome.com). In detail, eVai combines artificial intelligence with the American College of Medical Genetics (ACMG) guidelines (Richards et al. 2015) to classify all the genomic variants detected.

In order to identify potentially disease-causative variants, several filters were applied. In particular, all the selected variants presented a quality score (QUAL) > 20 and Minor Allele Frequency (MAF) < 0.001. In addition, variants were excluded if they led to non-damaging synonymous amino acid substitutions or did not affect splicing or highly conserved residues. To evaluate the pathogenicity of the identified variants, several *in-silico* tools were employed, including PolyPhen-2 (Adzhubei et al. 2013), SIFT (Ng and Henikoff 2003), Pseudo Amino Acid Protein Intolerance Variant Predictor (for coding variants SNVs/INDELs) (PaPI score) (Limongelli et al. 2015), and Deep Neural Network Variant Predictor (for coding/non-coding variants, SNVs) (DANN score) (Quang et al. 2015). In conclusion, the correlation between the variants and the phenotypes was discussed on a patient-by-patient basis, and the related literature was evaluated. The most compelling variants were confirmed by direct Sanger sequencing.

### Cell culture

Human embryonic kidney (HEK) 293 Phoenix and HeLa cells were used for cell-based assays. Details on cell culturing are given in the Additional file 2.

### Plasmid constructs

The pTARGET (Promega Corporation, Madison, WI, United States) vector contained the open reading frame (ORF) coding for wild-type human SLC26A4 (NCBI Sequence ID: NM_000441.2) with a hexahistidine tag at the C-terminus. The ORF of *TJP2* (NCBI Sequence ID: NM_004817.4) was subcloned into the pEYFPN1 and pECFPN1 vectors (Clontech Laboratories Inc., Mountain View, CA, United States) by PCR amplification of the pLDNT7_nFLAG DNASU clone HsCD00617962 (Seiler et al. 2014). The pEYFPN1 and pECFPN1 vectors encode for the protein of interest (SLC26A4 or TJP2) with the enhanced yellow fluorescent protein (EYFP) or the enhanced cyan fluorescent protein (ECFP) fused at the C-terminus.

Sequence alterations in plasmid vectors were made using the QuikChange^®^ site-directed mutagenesis kit (Agilent, Santa Clara, CA, United States) according to the manufacturer’s instructions and the primers listed in Additional file 2: Table S2. All plasmid inserts were sequenced before use in experiments (Microsynth AG, Balgach, Switzerland).

### SLC26A4 ion transport measurements

HEK 293 Phoenix cells were seeded into black 96-well plates, grown overnight, and co-transfected with 0.12 μg/well of a plasmid encoding for the iodide-sensitive EYFP variant p.H148Q;I152L (Galietta et al. 2001) and 0.12 μg/well of a pTARGET plasmid encoding for wild-type SLC26A4 or its variants by the calcium phosphate co-precipitation method. The endogenous iodide influx was determined in cells co-transfected with 0.12 μg/well of the EYFP p.H148Q;I152L vector and 0.12 μg/well of the empty pTARGET vector. Controls for the endogenous and the wild-type SLC26A4 iodide influx were included in each plate, along with the different SLC26A4 variants tested. The background fluorescence was measured in cells transfected with 0.24 μg/well of the pTARGET vectors.

The ion transport function of SLC26A4 was measured 48 h after transfection via a fluorometric method that allows for the evaluation of the iodide influx in SLC26A4-transfected cells (Roesch et al. 2018; Matulevicius et al. 2022; Procino et al. 2013; Pera et al. 2008a; Fugazzola et al. 2007; Dror et al. 2010; Dossena et al. 2011a; Dossena et al. 2011b; Dossena et al. 2006; de Moraes et al. 2016; Bernardinelli et al. 2016). Details are given in the Additional file 2.

### Determination of SLC26A4 protein expression levels by quantitative imaging

HeLa cells were seeded into six-well plates, grown overnight to approximately 50% confluence, and transiently transfected with 1.5 μg/well of the pEYFPN1-SLC26A4 vector and 3 μl METAFECTENE PRO^®^ (Biontex, Munich, Germany), following the manufacturer’s instructions. This vector encodes SLC26A4 with the enhanced yellow fluorescent protein (EYFP) fused to its C-terminus (SLC26A4-EYFP). The medium was replaced 6–8 h after transfection, and cells were transferred on glass slides 56 h after transfection and processed 72 h after transfection.

Quantitative imaging was done as formerly described (Roesch et al. 2018; Matulevicius et al. 2022; de Moraes et al. 2016). Shortly, cells were fixed with 4% paraformaldehyde for 30 min, counterstained with 0.1 μg/mL 4′,6-diamidino-2-phenylindole (DAPI) for 10 min, thoroughly washed and imaged in Hank’s balanced salt solution (HBSS, Sigma-Aldrich). Imaging was performed with a Leica TCS SP5II AOBS confocal microscope (Leica Microsystems, Wetzlar, Germany) equipped with an HCX PL APO 63x/1.20 Lambda blue water immersion objective and controlled by the LAS AF SP5 software version 2.7.3.9723 (Leica Microsystems). Imaging parameters are given in the Additional file 2.

### Colocalization

HEK 293 Phoenix cells were seeded on 3 cm diameter glass slides into 6-well plates, grown overnight, and transfected with 2 μg/well of the pEYFPN1 plasmid encoding for wild-type TJP2 or TJP2 variant p.T636R by the calcium phosphate co-precipitation method.

The impact of the variant on TJP2 subcellular localization was determined in live HEK 293 Phoenix cells by co-localization of wild-type or mutant TJP2-EYFP and the plasma membrane, which was stained with 1.25 μg/ml CellMask™ Deep Red Plasma Membrane Stain (C10046, Invitrogen Molecular Probes, Waltham, MA, USA), as formerly described (Matulevicius et al. 2022; de Moraes et al. 2016). Details are given in the Additional file 2.

### Fluorescence resonance energy transfer

HEK 293 Phoenix cells were seeded on 3 cm diameter glass slides into 6-well plates, grown overnight, and transfected with the pEYFPN1 and pECFPN1 plasmids (1 μg/well each) encoding for wild-type TJP2 or its variant by the calcium phosphate co-precipitation method. These vectors encode the proteins of interest with the fluorescence resonance energy transfer (FRET) donor ECFP or the FRET acceptor EYFP fused to their C-terminus. Thirty hours post-transfection, cells were fixed with 4% paraformaldehyde in HBSS for 30 min, and imaging was performed by sequential acquisition in HBSS at room temperature with the FRET AB-Wizard of the LAS AF SP5 software (Leica Microsystems) and a Leica TCS SP5II AOBS confocal microscope (Leica Microsystems). Acquisition and acceptor photobleaching parameters are given in the Additional file 2.

### Circulating nucleic acids

Peripheral blood samples (10 ml) were taken from each donor for 3 consecutive days and centrifuged at 1900 × *g* for 10 min at 4 °C. The plasma supernatant was collected and centrifuged again at 16,000 × *g* for 10 min at 4 °C to minimize contamination from cellular debris. Circulating nucleic acids (CNAs) were immediately extracted from the supernatant with the QiaAmp Circulating Nucleic Acids Kit (Qiagen) and eluted in 30 μl buffer AVE. Each sample was subjected to 2 independent reverse transcriptions with the QuantiTect^®^ reverse transcription kit for cDNA synthesis with integrated removal of genomic DNA contamination (Qiagen). Total RNA from human whole kidney cortex was from Ambion (Foster City, CA, USA). PrimeTime^®^ qPCR primers for detecting the *SLC26A4* transcript and the housekeeping transcript *POLR2A* were from Integrated DNA Technologies (Coralville, IA, USA). qPCR reactions contained 5 µL of undiluted cDNA template in a 20 µL final volume of 1X primers and 1X GoTaq^®^ qPCR Master Mix (Promega, Madison, WI, USA). Real-time PCR reactions were performed in technical duplicates for each sample, along with a minus reverse transcriptase control and no template control on the Rotor-Gene (Qiagen) instrument. Transcript levels were normalized for those of the housekeeping transcript and analyzed with the comparative ΔCt method (Livak and Schmittgen 2001).

### Salt and chemicals

All salt and chemicals were of *pro analysis* grade.

### Statistical analysis

All data are expressed as arithmetic means ± standard error of the mean (S.E.M.). For statistical analysis and generation of graphics, GraphPad Prism (version 9.5.1 for Mac OS, GraphPad Software, San Diego, CA, United States) and Excel (Microsoft, Redmond, WA, United States) software were used. Significant differences between data sets were determined by the unpaired Student’s *t*-test or ANOVA with Bonferroni’s or Dunnet’s ad hoc post-test, or the Fisher exact test, as appropriate. Statistically significant differences were assumed at *p* < 0.05; (n) corresponds to the number of independent measurements.

## Results

### Clinical features of patients

Our cohort of 32 patients with hearing loss and EVA represents an expansion of an original cohort of which the clinical characteristics of patients 1–16 have been formerly described (Roesch et al. 2018). The demographics and clinical features of the newly recruited patients 17–32 are described in Table 1. Overall, patients had sensorineural (29/32, 91%), conductive (2/32, 6%), or mixed (1/32, 3%) hearing loss associated with bilateral (27/32, 84%) or unilateral (5/32, 16%) EVA with IP2 (11/32, 34.5%), IP3 (2/32, 6%), or a normal cochlea (19/32, 59.5%). Additional clinical features of hearing loss and vestibular dysfunction are presented in Additional file 2: Tables S3 and S4.

**Table 1:** Demographic data and clinical features of the newly recruited patients

Patient	Patient ID	Ethnicity	Sex	Age (years)	EVA, side	IP2, side	Endolymphatic sac enlarged	Side affected by HL	Type of HL
17	#653	Caucasian	Male	11	Bilateral	No	Yes, B	Bilateral	Sensorineural
18	#654	Caucasian	Female	8	Bilateral	Yes, B	Yes, B	Bilateral	Sensorineural
19	#657	Caucasian	Female	54	Bilateral	No	n/a	Unilateral, L	Sensorineural
20	#659	Caucasian	Male	42	Bilateral	Yes, B	n/a	Bilateral	Sensorineural
21	#660	Caucasian	Male	27	Bilateral	Yes, B	No	Bilateral	Sensorineural
22	#663	Caucasian	Male	44	Bilateral	No	No	Bilateral	Sensorineural
23	#666	Caucasian	Female	17	Bilateral	Yes, B	Yes, R	Bilateral	Conductive
24	#667	Caucasian	Male	21	Unilateral, L	No, but had IP3 B	No	Bilateral	Sensorineural
25	#669	Caucasian	Male	56	Bilateral	No	n/a	Bilateral	Sensorineural
26	#670	Caucasian	Female	7	Bilateral	Yes, B	Yes, B	Bilateral	Sensorineural
27	#671	Caucasian	Female	51	Bilateral	No	Yes, B	Bilateral	Sensorineural
28	#672	Caucasian	Male	8	Unilateral, L	No	Yes, L	Asymmetric	Sensorineural
29	#678	Caucasian	Female	51	Bilateral	Yes, B	n/a	Bilateral	Sensorineural
30	#679	Caucasian	Female	65	Bilateral	No	No	Bilateral	Sensorineural
31	#680	Caucasian	Male	54	Bilateral	No	No	Unilateral, R	Sensorineural
32	#681	Caucasian	Male	5	Bilateral	Yes, B	Yes, L	Bilateral	Sensorineural

B, bilateral, HL, hearing loss, IP2, cochlear incomplete partition type 2, IP3, cochlear incomplete partition type 3, L, left, n/a, not assessed, R, right

For a subset of patients, the thyroid function was tested with the perchlorate discharge test, which was negative (discharge < 15%) for patients #358, 659, and 671 and positive (discharge ≥ 15%) for patient #660. Patient #678 had hypothyroidism.

### Variant detection in formerly known EVA genes

Target amplification and Sanger sequencing were performed for genes formerly linked to non-syndromic EVA (*GJB2, SLC26A4, FOXI1, KCNJ10*, and *POU3F4*) and *GJB3*.

Concerning *GJB2*, a common sequence alteration (c.35delG) with established pathogenicity (Denoyelle et al. 1997) has been found in 3/32 (9%) patients. Of these, 2/32 (6%) patients harbored this variant in homozygosis, and one patient (1/32, 3%) harbored this variant in heterozygosis with the wild-type allele, which is a non-diagnostic genotype. The results of Sanger sequencing of *GJB2* are shown in Additional file 2: Table S5.

Concerning *SLC26A4*, the results of patients 1–16 are shown in our former work (Roesch et al. 2018). The comprehensive results of the newly recruited patients 17–32 are shown in Additional file 2: Table S6. In this last group, 10 *SLC26A4* potentially causative variants were found in 7 patients (Tables 2 and 3, patients 17–32). Of these, 8 are predicted to lead to an amino acid substitution, one affects splicing, and one leads to premature truncation of the protein product. Two variants (p.Q101R and p.N248Kfs*41) are novel, two (p.Y78C and p.I136N) are not reported in ClinVar, two (p.L597S and p.G740V) have conflicting classifications of pathogenicity in ClinVar (likely benign/uncertain significance/benign), and four (c.1001 + 1G > A, p.R185T, p.R409H, and p.A664V) were categorized as pathogenic or likely pathogenic. In addition, several variants in non-coding regions of *SLC26A4* were detected (Additional file 2: Table S6). In the whole cohort (Table 3), potentially causative biallelic variants in *SLC26A4* were detected in 5/32 patients (16%). Based on the positive perchlorate discharge test, one of these patients (1/5, 20%) had Pendred syndrome (patient #660). Monoallelic variants (non-diagnostic genotype) affecting the *SLC26A4* protein product were detected in 5/32 patients (16%).

**Table 2:** Pathogenicity assignment for all *SLC26A4* variants identified in the cohort

Patient ID	Gene	cDNA	Protein	SNP ID	Allele frequency	PaPI	PolyPhen-2	DANN	SIFT	Alpha missense	Clinical significance
#271	*SLC26A4*	c.1301C > A	p.A434D	rs1035397261	0.00000958	1 (damaging)	0.770 (possibly damaging)	0.9969 (damaging)	0.001 (damaging)	0.970 (likely pathogenic)	VUS
		c.1730 T > C	p.V577A	rs56017519	0.00000412	0.999 (damaging)	0.983 (damaging)	0.9989 (damaging)	0.003 (damaging)	0.8288 (likely pathogenic)	VUS
#358	*SLC26A4*	c.61A > G	p.M21V	rs375716219	0.0000254	0.0 (tolerated)	0.0 (benign)	0.4068 (likely benign)	0.982 (tolerated)	0.04619 (likely benign)	Likely benign
#616	*SLC26A4*	c.343 T > G	p.Y115D	NA	NA	0.981 (damaging)	0.979 (damaging)	na	0.11 (tolerated)	0.6443 (likely pathogenic)	VUS
#653	*SLC26A4*	c.2219G > T	p.G740V	rs111033310	0.000209	0.137 (tolerated)	0.002 (benign)	0.9899 (damaging)	0.007 (damaging)	0.08299 (likely benign)	VUS
#654	*SLC26A4*	c.1790 T > C	p.L597S	rs55638457	0.0086	0.995 (damaging)	0.987 (damaging)	0.9988 (damaging)	0.002 (damaging)	0.9147 (likely pathogenic)	VUS
#659	*SLC26A4*	c.407 T > A	p.I136N	rs1000929089	0.0000065	0.992 (damaging)	0.992 (damaging)	0.9937 (damaging)	0 (damaging)	0.7665 (likely pathogenic)	Likely pathogenic
		c.1991C > T	p.A664V	rs2129318281	0.00000137	0.936 (damaging)	0.930 (damaging)	0.991 (damaging)	0.07 (benign)	0.2116 (likely benign)	Likely pathogenic
#660	*SLC26A4*	c.302A > G	p.Q101R	NA	NA	0.998 (damaging)	0.998 (damaging)	NA	0 (damaging)	0.9592 (likely pathogenic)	VUS
		c.1226G > A	p.R409H	rs111033305	0.0000815	1 (damaging)	1 (damaging)	0.9994 (damaging)	0 (damaging)	0.9499 (likely pathogenic)	Pathogenic
#670	*SLC26A4*	c.554G > C	p.R185T	rs542620119	0.0000513	0.981 (damaging)	0.981 (damaging)	0.9815 (damaging)	0.001 (damaging)	0.9569 (likely pathogenic)	Pathogenic
		c.744delT	p.N248Kfs*41	NA	NA	1 (damaging)	NA	NA	NA	NA	VUS
#671	*SLC26A4*	c.1001 + 1G > A	splice donor	rs80338849	0.000298	NA	NA	0.9956 (Damaging)	NA	NA	Pathogenic
#681	*SLC26A4*	c.233A > G	p.Y78C	rs2129309178	0.00000205	1 (damaging)	1 (damaging)	0.9983 (damaging)	0 (damaging)	0.9642 (likely pathogenic)	Pathogenic
		c.233A > G	p.Y78C	rs2129309178	0.00000205	1 (damaging)	1 (damaging)	0.9983 (damaging)	0 (damaging)	0.9642 (likely pathogenic)	Pathogenic

Allele frequency according to gnomAD (Genome Aggregation Database), the pathogenicity score according to 5 prediction tools (PaPi: http://papi.unipv.it/, PolyPhen-2: http://genetics.bwh.harvard.edu/pph2/, DANN: https://pubmed.ncbi.nlm.nih.gov/25338716/, SIFT: https://sift.bii.a-star.edu.sg/, AlphaMissense: https://alphamissense.hegelab.org/, all accessed on 30.08.2024) and the clinical significance according to the ACMG and ClinGen recommendations. NA, not assessed; VUS, variant with uncertain significance

**Table 3:**
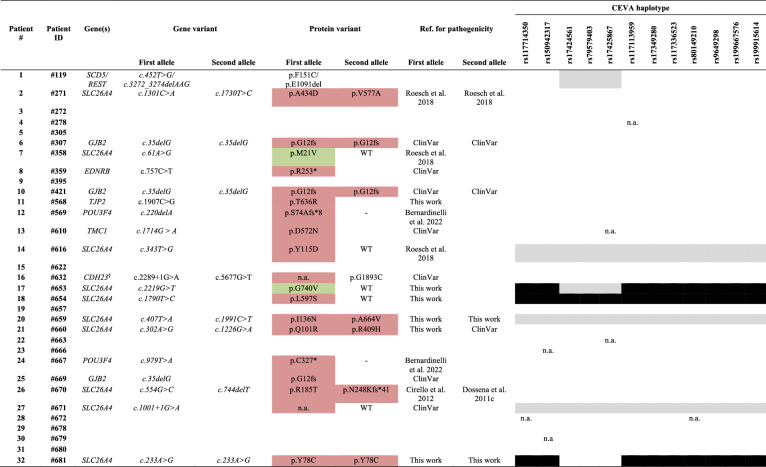
Gene variants putatively causing hearing loss in the EVA cohort

Gene variants reported in ClinVar as pathogenic or benign, or for which the pathogenicity was confirmed or excluded by functional testing are indicated in red and green, respectively. References to these functional studies are also given. Pathogenicity of the novel pendrin variant p.N248Kfs*41 was inferred based on the fact that all pendrin truncations tested exhibited loss of function (Dossena et al. 2011c). Monoallelic and biallelic CEVA individual SNPs are indicated in grey and black, respectively. Reference sequences are as follows: for *GJB2*, NM_004004.6; for *SLC26A4*, NM_000441.2; for *POU3F4*, NM_000307.1; for *TMC1*, NM_138691.3. The reference sequences of the other genes are reported in Table 4. CEVA, Caucasian EVA haplotype. n.a., not assessed. § phase unknown

The results of Sanger sequencing of *FOXI1* are shown in Additional file 2: Table S7. The great majority of variants detected were established benign variants or intronic variants not reported in ClinVar. Exonic synonymous monoallelic variants with conflicting pathogenicity classifications were detected in patients #307 and #678. The results of Sanger sequencing of *KCNJ10* are shown in Additional file 2: Table S8. Exonic variants with conflicting classifications of pathogenicity leading to an amino acid substitution were detected in patients #616 and #660. The results of Sanger sequencing of *GJB3* are shown in Additional file 2: Table S9. Only established benign variants were found in this gene. The results of Sanger sequencing of *POU3F4* are shown in Additional file 2: Table S10. Exonic variants leading to protein truncation were detected in 2/32 patients (6%). These variants have been established as pathogenic in our former work (Bernardinelli et al. 2022). No CNV of the *SLC26A4* and *STRC* genes or genomic deletions del(GJB6-D13S1830) and del(GJB6-D13S1854) were detected in this cohort. Potentially causative sequence alterations leading to an amino acid change, frameshift/truncation of the protein product, or affecting splicing in the above-mentioned genes are reported in Table 3.

### Pathogenicity assignment of SLC26A4 variants leading to an amino acid substitution

**Fig. 1:**
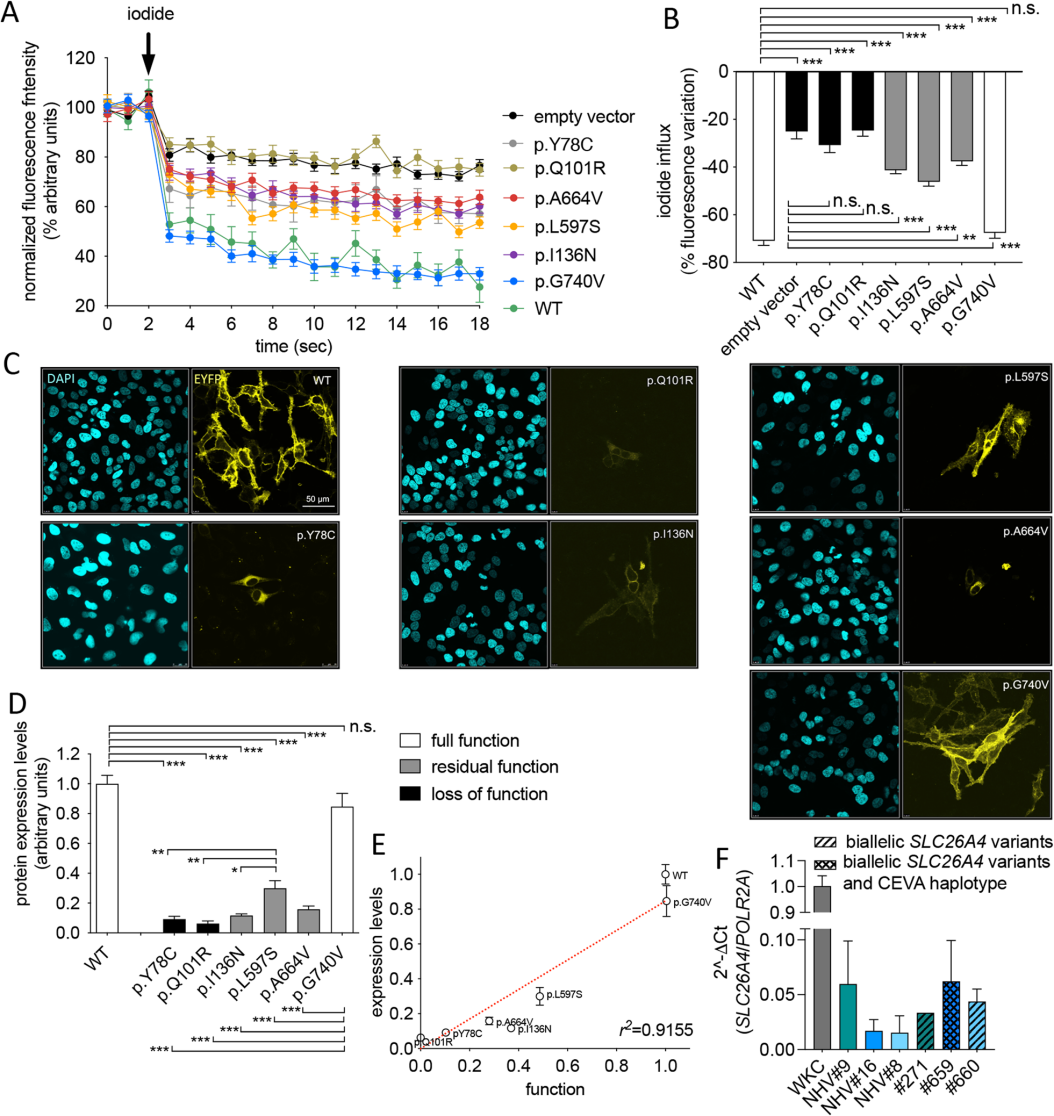
Ion transport function and expression of pendrin variants. **A** HEK 293 Phoenix cells were co-transfected for 48 h with plasmid vectors encoding the wild-type or mutant pendrin or an empty vector and the iodide sensor EYFP H148Q:I152L. Ion transport activity was determined with a fluorometric method by measuring the intracellular fluorescence over time before and after the addition of iodide to the extracellular solution (arrow). **B** Iodide influx expressed as the % decrease of the intracellular fluorescence. 17 ≤ n ≤ 37 from at least 3 independent experiments. *n* corresponds to an individual well of a 96-well plate and coincides with the number of independent transfections. **C** HeLa cells were transfected for 72 h with plasmid vectors encoding wild-type or mutant SLC26A4-EYFP (yellow), fixed, counterstained with DAPI (cyan), and imaged. Scale bar 50 µm. **D** Total expression levels of pendrin variants were determined by quantitative imaging and normalized for those of the wild-type. 12 ≤ n ≤ 18 from 3 independent experiments. *n* correspond to the imaging fields. n.s., not statistically significant, ***p < 0.001, **p < 0.01, *p < 0.05 one-way ANOVA with Bonferroni’s post-test. **E** Ion transport function (iodide influx) and protein abundance of pendrin variants were expressed as % of the wild-type and positive correlation between data sets was tested by linear regression. The r^2^ value is indicated. **F** Transcript levels of *SLC26A4* measured by RT-qPCR in circulating nucleic acids from 3 patients and 3 ethnicity, sex, and age-matched normal hearing volunteers (NHV). The color code indicates matched individuals. Human whole kidney cortex (WKC) served as a control of primer efficiency. Data are from 3 blood samples from each subject, except for patient #271, for whom only one blood sample was available. No statistically significant differences among data sets were found (one-way ANOVA with Bonferroni’s post-test)

SLC26A4 protein variants p.Y78C, p.Q101R, p.I136N, p.L597S, p.A664V, and p.G740V have been selected for cell-based assays. Ion transport activity was determined as the efficiency of iodide influx in transfected cells, and results were compared to the ion transport efficiency in cells transfected with wild-type pendrin or no pendrin (empty vector, Fig. 1a, b). Ion transport of variants p.Y78C and p.Q101R were significantly reduced compared to the wild-type and indistinguishable from the empty vector, and these were classified as variants with loss of function. Ion transport of variants p.I136N, p.L597S, and p.A664V was reduced compared to the wild-type but significantly higher than the empty vector, and these were classified as variants with residual function. Ion transport of variant p.G740V was indistinguishable from the wild-type and this was classified as a variant with full function. Reduction in ion transport function of the different variants unlikely arose from a reduction in the transfection efficiency of the corresponding plasmid constructs, as differences between the transcript levels of wild-type and variant *SLC26A4* were never detected in our former studies (Matulevicius et al. 2022; de Moraes et al. 2016).

The protein expression levels of these variants were evaluated by quantitative imaging in transfected cells. Consistent with functional studies, the protein levels of variant p.G740V were not significantly reduced compared to the wild-type. In contrast, all of the functionally affected variants showed reduced protein levels (Fig. 1c, d), supporting their pathogenicity. There was a positive correlation between ion transport function and protein levels (Fig. 1e).

Based on these results, benign and pathogenic pendrin variants are indicated in green and red in Table 3, respectively. Pathogenicity of pendrin variants p.M21V, p.Y115D, p.A434D, and p.V577A was assigned or excluded based on functional testing in our former study (Roesch et al. 2018).

### Detection of the CEVA haplotype

The possible presence of all 12 SNPs of the CEVA haplotype was verified by SNP assay or Sanger sequencing in all patients (Table 3). Within the largest region of linkage disequilibrium, a sub-region of three SNP (rs17424561, rs79579403, and rs17425867) segregated independently from the other nine SNPs in three patients (#119, 653, and 681). The shorter version of the CEVA haplotype comprising the nine telomeric variants located in their own region of higher linkage disequilibrium was formerly described by Chattaraj et al. in one patient and by Smits et al. in two patients and was suggested to represent the true pathogenic CEVA haplotype (Chattaraj et al. 2017; Smits et al. 2022). The genetic configuration of the CEVA haplotype was complex. Part or the entire haplotype was detected in 7/32 patients (22%) as monoallelic or biallelic variants. Three of these patients (#616, 654, and 671) harbor monoallelic pathogenic sequence alterations in the pendrin gene. Of these patients, #616 and 671 harbor the CEVA haplotype as monoallelic variants. In these two patients, the CEVA haplotype would be causative if *in trans* with the monoallelic pathogenic pendrin variant. Therefore, segregation studies were conducted in these two patients. Unfortunately, it was impossible to unequivocally establish whether the CEVA haplotype was *in trans* with the pathogenic *SLC26A4* variant in these patients (Additional file 2: Fig. S1 and S2).

Segregation studies conducted in the family of patient #653 showed that this patient inherited the CEVA haplotype and the benign SLC26A4 variant p.G740V from the father and a partial CEVA haplotype from the mother (Additional file 2: Fig. S3). Patient #659 inherited the pathogenic SLC26A4 variant p.A664V from the father and the pathogenic SLC26A4 variant p.I136N along with the CEVA haplotype from the mother (Additional file 2: Fig. S4). These segregation studies indicate that the CEVA haplotype is often found *in cis* with *SLC26A4* variants.

### Detection of SLC26A4 transcript in CNAs

*SLC26A4* transcript abundance in CNAs was measured in the peripheral blood of three patients with biallelic pathogenic pendrin variants with or without the CEVA haplotype and three ethnicity, sex, and age-matched normal hearing volunteers (Fig. 1f). Although the *SLC26A4* transcript was reproducibly detected in CNAs, no differences between transcript levels have been observed among study subjects.

### Whole exome sequencing

Selected patients with bilateral EVA and negative for known causative genes (patients #119, 305, 359, 568, 610, 622, 632, 663, 669, 678, and 679, 11/32, 34%) have been re-analyzed by ES. ES detected 7 variants in 6 genes (*SCD5, REST, EDNRB, TJP2, TMC1*, and *CDH23*) formerly unrelated to EVA in 5/32 patients (16%). *TMC1* variant p.D572N was described in our former study (Frohne et al. 2024) and is categorized as pathogenic in ClinVar. The results of this study are shown in Table 4. Of the 6 variants detected, one within *CDH23* (c.5677G > T; p.G1893C) is novel (no SNP ID is assigned), while two, within the *CDH23* (c.2289 + 1G > A) and *EDNRB* (c.757C > T; p.R253*) genes are established pathogenic variants.

**Table 4:** Results of ES analysis

Patient ID	Gene	Ref. seq.	Chr position (assembly: GrCh37)	Genotype	cDNA	Protein	SNP ID	Allele frequency	PaPI	PolyPhen-2	DANN	SIFT	AlphaMissense	Clinical significance
#119	*REST*	NM_005612.5	Chr4:57798292	Het	c.3272_3274delAAG	p.E1091del	rs767853298	0.00001428	0.844 1 (damaging)	NA	NA	NA	NA	VUS
	*SCD5*	NM_001037582.3	Chr4:83601977	Het	c.452T > G	p.F151C	rs138660783	0.0002752	1 (damaging)	0.998 (damaging)	0.995 (damaging)	0 (damaging)	0.723 (likely pathogenic)	VUS
#359	*EDNRB*	NM_001122659.3	Chr13:78477335	Het	c.757C > T	p.R253*	rs104894390	0.000002480	0.996 (damaging)	NA	0.995 (damaging)	NA	NA	Pathogenic/likely pathogenic
#568	*TJP2*	NM_004817.4	Chr9:71851070	Het	c.1907C > G	p.T636R	rs1830172525	NA	1 (damaging)	1 (damaging)	0.995 (damaging)	0 (damaging)	0.154 (likely benign)	VUS
#632	*CDH23*^§^	NM_022124.6	Chr10:73454017	Comp het	c.2289 + 1G > A	NA	rs769433759	0.00001305	NA	NA	0.995 (damaging)	NA	NA	Pathogenic
			Chr10:73544822		c.5677G > T	p.G1893C	NA	NA	1 (damaging)	1 (damaging)	0.997 (damaging)	0 (damaging)	0.935 (likely pathogenic)	VUS

The allele frequency according to the GnomAD database (Chen et al. 2024), the pathogenicity score according to five *in-silico* prediction tools, and the variants classification according to the ACMG criteria (Richards et al. 2015) are reported. Comp het, compound heterozygous, Het, heterozygous with the wild-type allele, NA, not available, VUS, variant of uncertain significance. § phase unknown

### Pathogenicity assignment of the TJP2 variant p.T636R

**Fig. 2:**
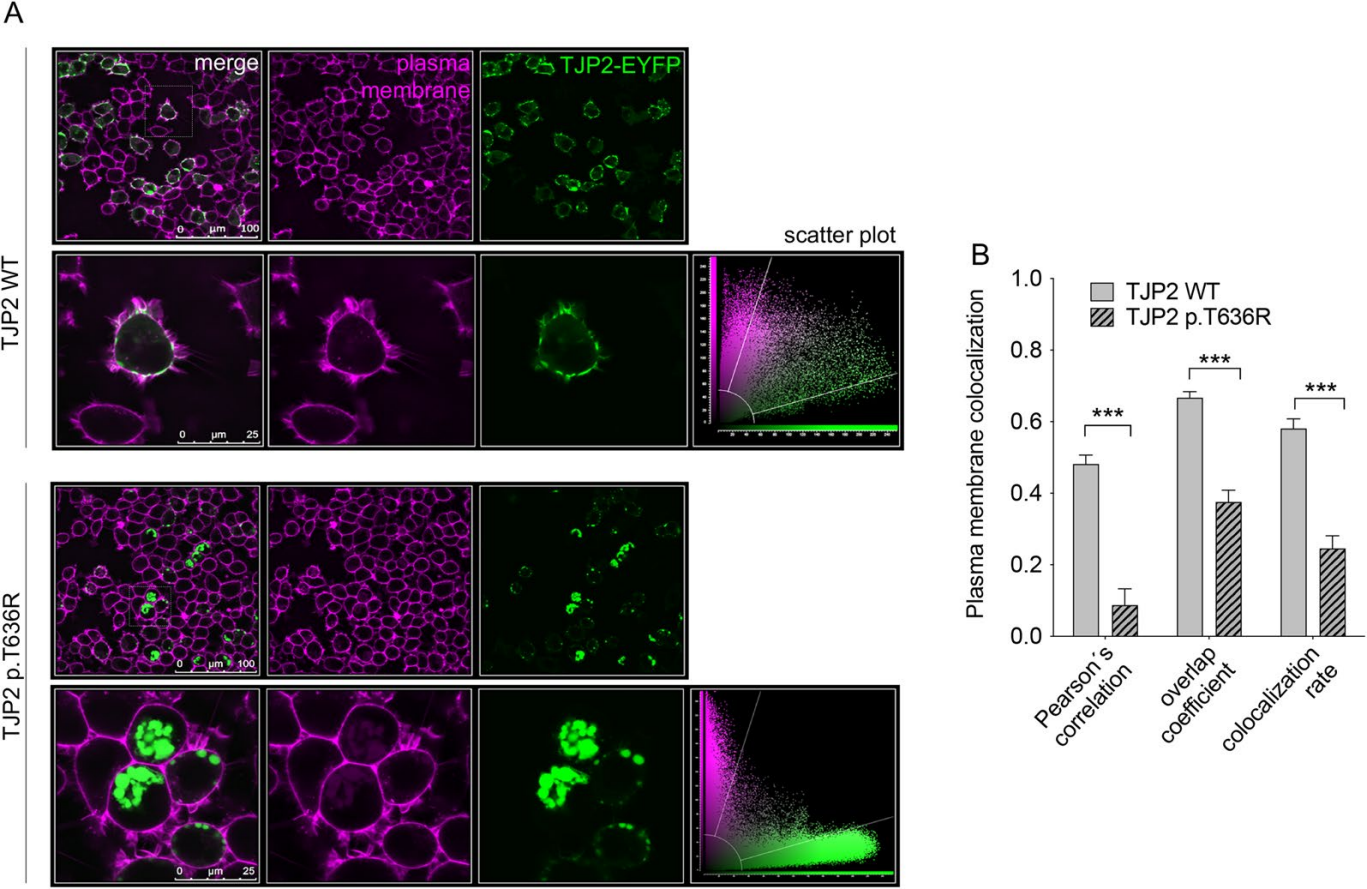
Subcellular localization of the TJP2 protein variant p.T636R. **A** Confocal images of live HEK 293 Phoenix cells transfected with wild-type TJP2 or TJP2 variant p.T636R (green). The subcellular localization was assessed 30 h after transfection by co-localization with the plasma membrane (magenta). The corresponding merge images and scatter plots are also shown. White pixels in the merge images indicate co-localization between the two signals. **B** Plasma membrane colocalization parameters of wild-type TJP2 or TJP2 variant p.T636R. 20 ≤ n ≤ 24 from 3 independent experiments. *n* corresponds to the number of cells. ***p < 0.001, unpaired, two-tailed Student’s *t*-test

Variant c.1907C > G in the tight-junction protein 2 (*TJP2*) gene leading to the amino acid substitution p.T636R in patient #568 was uncharacterized and therefore was selected for further analysis to assign or exclude its pathogenicity. The protein variant was ectopically expressed in cells and its subcellular distribution was analyzed and compared to the wild-type (Fig. 2). For this, the co-localization with the plasma membrane was determined. Wild-type TJP2 was uniformly distributed at the cell periphery and gave plasma membrane co-localization parameters (Pearson's correlation coefficient, overlap coefficient, and co-localization rate) consistent with a localization in the sub-membrane protein network. In contrast, p.T636R TJP2 formed large intracellular aggregates in approximately 50% of transfected cells and failed to reach the cell periphery.

**Fig. 3:**
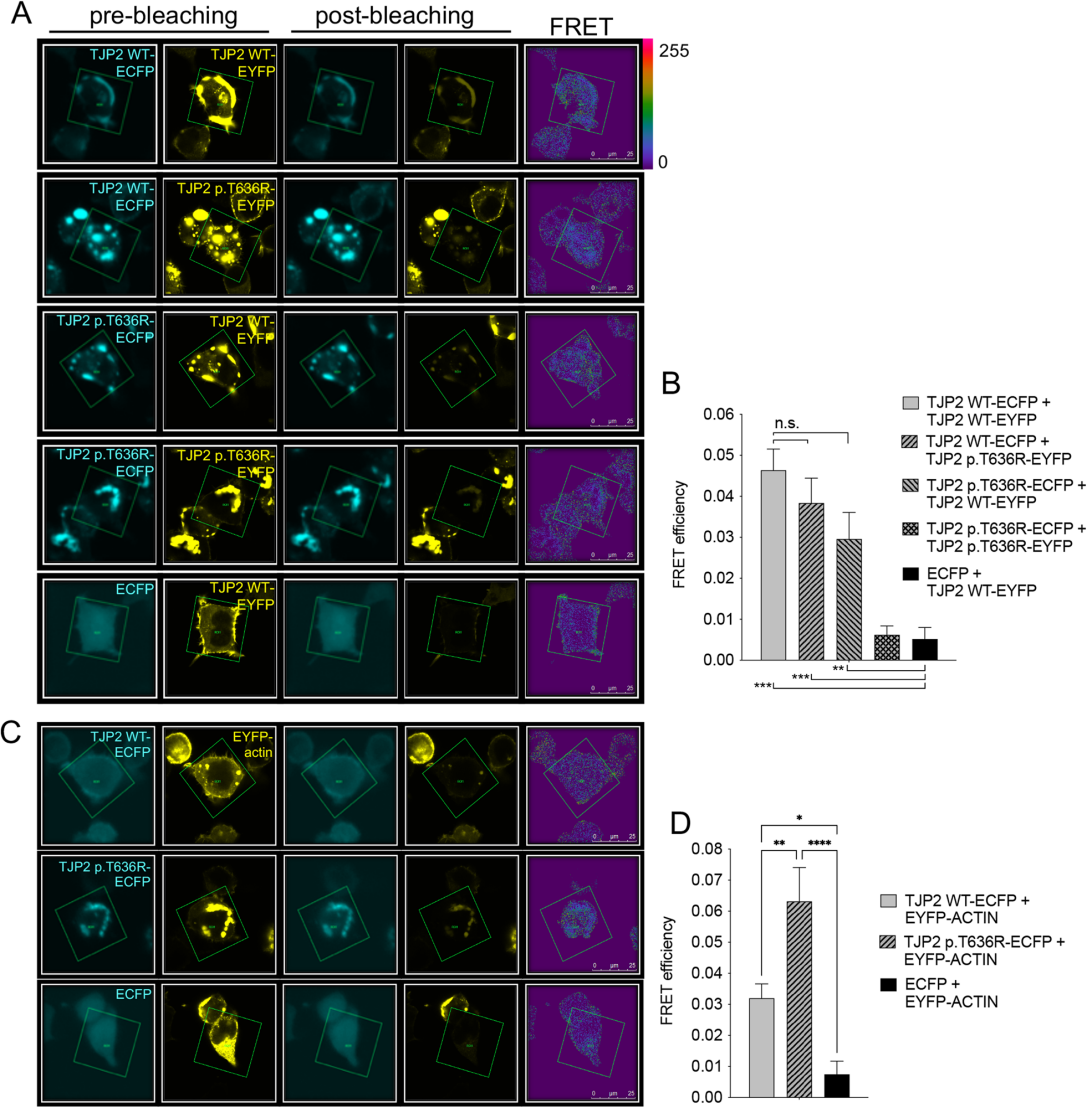
Dimerization of the TJP2 protein variant p.T636R and interaction with actin. **A** Fixed HEK 293 Phoenix cells transfected with wild-type TJP2 or TJP2 variant p.T636R with the FRET donor ECFP or the FRET acceptor EYFP fused to their C-terminus imaged before and after photobleaching of the acceptor and corresponding FRET image. **B** FRET efficiency of the indicated FRET pairs was determined to assess the homo- or heterodimerization of wild-type TJP2 or TJP2 variant p.T636R. Cells transfected with ECFP and TJP2 wild-type-EYFP served as the negative control. 18 ≤ n ≤ 21 from 3 independent experiments ***p < 0.001, **p < 0.01, one-way ANOVA with Bonferroni’s post-test. **C** Fixed HEK 293 Phoenix cells transfected with wild-type TJP2 or TJP2 variant p.T636R with the FRET donor ECFP fused to their C-terminus and actin with the FRET acceptor EYFP fused to the N-terminus imaged before and after photobleaching of the acceptor and corresponding FRET image. **D** FRET efficiency of the indicated FRET pairs was determined to assess the interaction of wild-type TJP2 or TJP2 variant p.T636R and actin. Cells transfected with ECFP and EYFP-actin served as the negative control. 23 ≤ n ≤ 25 from 4 independent experiments ****p < 0.0001, **p < 0.01, *p < 0.05, one-way ANOVA with Bonferroni’s post-test. *n* corresponds to the number of cells

The ability of wild-type and p.T636R TJP2 to form homo- and heterodimers and interact with actin was determined by FRET (Fig. 3). As expected, wild-type TJP2 formed homodimers, as documented by a significant FRET between the FRET pair wild-type TJP2-ECFP and wild-type TJP2-EYFP. Surprisingly, p.T636R TJP2 affected the plasma membrane localization of the co-expressed wild-type form. Accordingly, a significant FRET between the FRET pairs wild-type TJP2-ECFP and p.T636R TJP2-EYFP as well as wild-type TJP2-EYFP and p.T636R TJP2-ECFP was measured, consistent with the formation of wild-type TJP2/p.T636R TJP2 heterodimers. In contrast, p.T636R TJP2 could not form homodimers (Fig. 3a, b). As expected, wild-type TJP2 established a direct molecular interaction with actin, which, surprisingly, was also observed and was even stronger for p.T636R TJP2 (Fig. 3c, d).

## Discussion

**Fig. 4:**
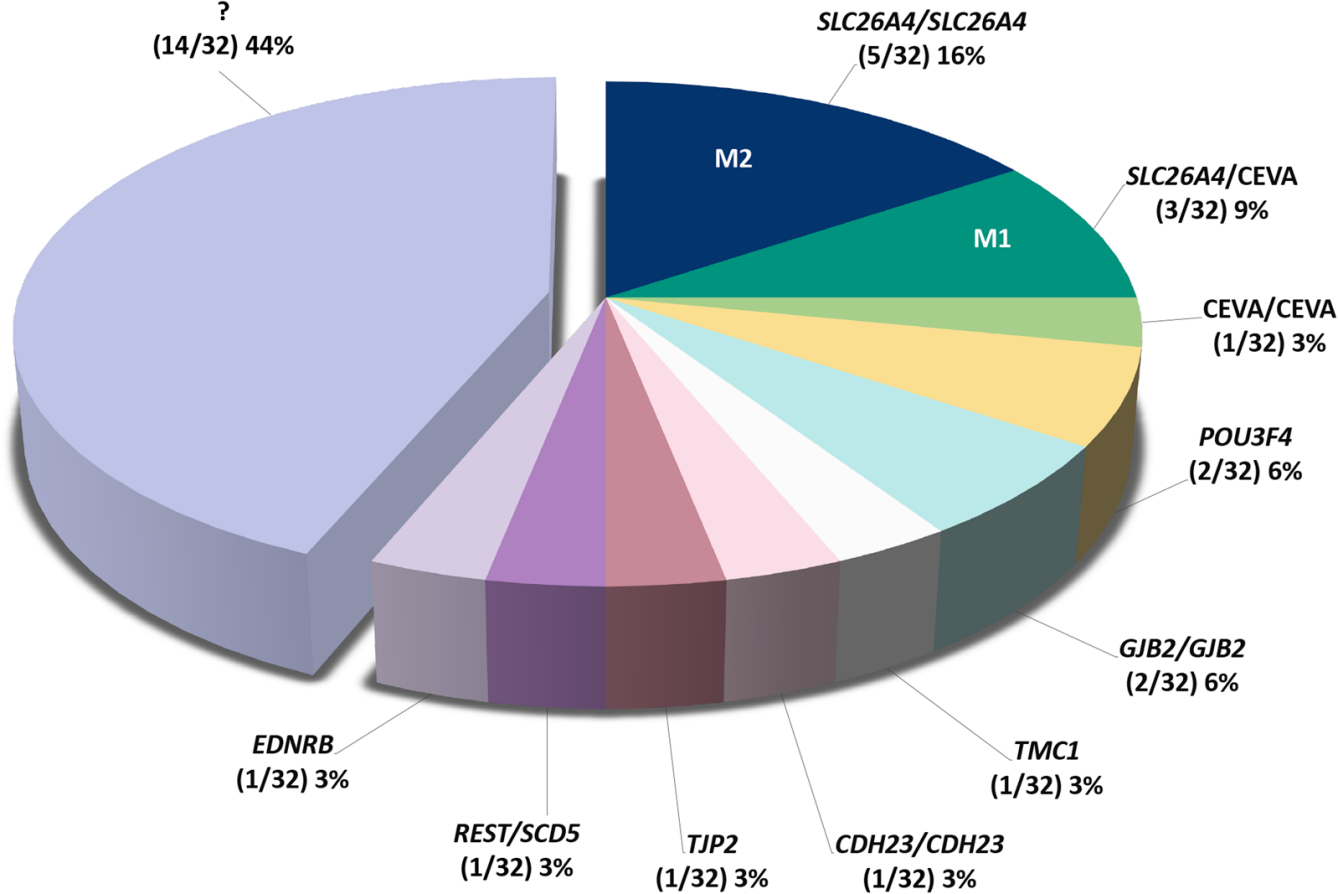
Genetic determinants of hearing loss and EVA in the Austrian cohort of 32 patients. Causative genes are indicated as first allele/second allele. In total, 18/32 patients (56%) have been diagnosed. CEVA, Caucasian EVA haplotype; M1, monoallelic pathogenic *SLC26A4* variants; M2, biallelic pathogenic *SLC26A4* variants. ? indicates 14/32 patients (44%) who remained undiagnosed

Functional studies of the protein product are essential in assigning or excluding the pathogenicity of a given gene variant. Concerning *SLC26A4*, in our former studies we have observed that reduction of expression is consistently observed for all protein variants with a reduction of function, regardless of subcellular distribution (Roesch et al. 2018; Matulevicius et al. 2022; de Moraes et al. 2016). In the newly recruited patients 17–32 of our cohort, 10 *SLC26A4* variants predicted to affect the protein product were found in 7 patients (Tables 2 and 3). Of these, one affects splicing, one leads to premature truncation of the protein, and 8 lead to an amino acid substitution. The splicing variant (c.1001 + 1G > A) is an established pathogenic variant frequent in patients of North European descent (Smith et al. 1993). Pathogenicity of the truncating variant p.N248Kfs*41 was inferred based on the observation that all pendrin truncations exhibit loss of function (Dossena et al. 2011c). Of the 8 *SLC26A4* variants leading to amino acid substitutions, two (p.R185T and p.R409H) were characterized by functional testing in former studies, supporting their pathogenicity (Cirello et al. 2012; Chattaraj et al. 2013; Gillam et al. 2005; Wasano et al. 2020). Variant p.A664V was formerly characterized via [^14^C] formate uptake studies and exhibited reduced function (Yuan et al. 2012). As formate may not be a physiological anion for pendrin, this variant was selected for functional testing in this study. Variant p.L597S gave inconsistent results in former studies in terms of functionality (Pera et al. 2008a; Choi et al. 2009) and was therefore included in this study. For the other four variants (p.Y78C, p.Q101R, p.I136N, and p.G740V), no functional studies are available in the literature and were therefore all included in this study. Based on expression levels and ion transport function in heterologous expression systems significantly reduced compared to the wild-type, variants p.Y78C, p.Q101R, p.I136N, p.L597S, and p.A664V have been classified as pathogenic (Fig. 1). In contrast, p.G740V was indistinguishable from the wild-type and was categorized as non-pathogenic (Fig. 1). These functional tests, together with those reported in the literature and our former study (Roesch et al. 2018), explain the phenotype of patients #271, 659, 660, 670, and 681, who harbor biallelic pathogenic pendrin variants (Table 2). All of them had bilateral EVA and an IP2 (Mondini malformation). Therefore, the genetic configuration M2 (biallelic pathogenic *SLC26A4* sequence alterations) was found in 5/32 (16%) of patients (Fig. 4) of our cohort, consistent with other Caucasian cohorts with hearing loss and EVA, where biallelic pendrin sequence alterations are found in approximately 25% of patients (Ito et al. 2013). Thus, the genetic configuration M2 explains hearing loss and EVA in only a fraction of patients. Consequently, the clinical finding of an EVA in the context of hearing loss does not unequivocally imply biallelic pathogenic sequence alterations in *SLC26A4*, providing a strong imperative for genetic testing to offer the correct diagnosis and the best possible patient care.

The CEVA haplotype can act as a recessive allele and explain EVA and hearing loss in patients with monoallelic pathogenic pendrin variants and possibly some patients with no pendrin variants (Chattaraj et al. 2017). Whether and to what extent the CEVA haplotype might also contribute to the phenotype in patients with pathogenic biallelic pendrin variants (#659 and 681) is unclear. In our cohort, the biallelic CEVA haplotype was found in patient #653, who harbors the monoallelic non-pathogenic pendrin variant p.G740V, in patient #654, who harbors the monoallelic pathogenic pendrin variant p.L597S and in patient #681, harboring the pathogenic biallelic p.Y78C pendrin variant (Table 3). In patients #616 and 671, a monoallelic CEVA haplotype was identified. These patients harbor monoallelic pathogenic pendrin variants, it is therefore essential to determine whether the pendrin variant lies on the same allele as the CEVA haplotype. Unfortunately, this could not be unequivocally assessed by our segregation studies (Additional file 2: Fig. S1 and S2). However, it appears that the CEVA haplotype is frequently found on the same allele as a pendrin variant (Additional file 2: Fig. S1–S4). These findings suggest caution and precise segregation studies when attributing causality to the CEVA haplotype. Assuming that the CEVA haplotype is causative, it can explain the phenotype in M1 (monoallelic pathogenic *SLC26A4* sequence alterations) patients #616, 654, and 671 (Table 3), that is 3/22 (9%) patients of the cohort (Fig. 4). Assuming that *SLC26A4* p.G740V variant is benign (Table 3), the biallelic CEVA haplotype alone could have been causative in patient #653, that is 1/22 (3%) patients of the cohort (Fig. 4).

Concerning the p.G740V variant, this variant is tolerated according to 3 out of 5 pathogenicity prediction tools (Table 2) and was recently re-categorized as VUS (Quaio et al. 2022). Glycine residue at position 740 is not conserved in SLC26A4 orthologues and variant p.G740S had no reduced function according to two distinct functional tests (Dossena et al. 2011a). Thus, amino acid substitutions at position 740 seem to be tolerated. Curiously, variant p.G740V was found *in cis* with variant p.T307M in two independent reports (Pera et al. 2008b; Albert et al. 2006). p.T307M is a VUS according to ClinVar. In both patients, there was another pathogenic variant *in tran*s. Therefore, it is uncertain whether p.G740V, p.T307M, or both, were causative in these patients. p.G740V variant was also found in a patient with severe congenital hypothyroidism, with no mention of hearing loss (Makretskaya et al. 2018). Interestingly, there is another report where the p.G740V variant was found with the homozygous CEVA haplotype (Baldyga et al. 2023), similar to what we have found in patient #653 (Table 3). The homozygous CEVA haplotype was also described in association with variant p.M775T (Chattaraj et al. 2017). Variant p.M775T is hypofunctional (Choi et al. 2009), and could have been causative. Variant p.G740V, however, is fully functional according to our results (Fig. 1), giving supporting evidence for non-pathogenicity. Thus, we suggest that either the homozygous CEVA haplotype or other undermined genetic or environmental factors could have been causative in patient #653.

For pinpointing the pathogenic effect of the CEVA haplotype, investigations addressing defect(s) at the RNA, protein, or epigenetic level are required (Smits et al. 2022). To verify the hypothesis that the CEVA haplotype may alter a *SLC26A4* regulatory region and affect its transcription, the *SLC26A4* transcript abundance in CNAs was measured as a proxy of tissue abundance. CNAs contain protein-coding cell-free mRNA (Pos et al. 2018). The only CEVA patient available for this investigation was patient #659, who harbored a monoallelic CEVA haplotype and biallelic pathogenic *SLC26A4* sequence alterations. Therefore, patients with biallelic pathogenic *SLC26A4* sequence alterations but no CEVA haplotype and ethnicity, sex, and age-matched normal hearing volunteers have been recruited as controls. *SLC26A4* transcript was reproducibly detected in CNAs, consistent with its abundant expression in highly perfused organs such as the kidney and thyroid. If the CEVA haplotype falls in a *SLC26A4* regulatory region and impairs its transcription, we would expect the *SLC26A4* transcript levels in CNAs from patient #659 to be reduced compared to those of patients with no CEVA haplotype or normal hearing volunteers. However, no reduction in *SLC26A4* transcript abundance in CNAs could be observed in patient #659, therefore the hypothesis of an impaired *SLC26A4* transcription could not be supported (Fig. 1f). Also, no reduction in *SLC26A4* transcript levels could be detected in patients with biallelic *SLC26A4* variants and no CEVA haplotype compared to normal hearing controls, consistent with our hypothesis that reduction in transcript levels plays no role in the loss of function of pathogenic protein variants of *SLC26A4.* Instead, loss of function of pathogenic protein variants of *SLC26A4* likely arises from a reduced protein expression (Fig. 1c, d) (Matulevicius et al. 2022; de Moraes et al. 2016), and probably stems from increased protein degradation.

Recessive digenic inheritance of EVA caused by a mutation in *SLC26A4* and another mutation in *FOXI1* or *KCNJ10* has also been suggested (Yang et al. 2007; Yang et al. 2009). Biallelic pathogenic variants in *FOXI1* cause early-onset sensorineural deafness and distal renal tubular acidosis (Enerback et al. 2018) and *FOXI1* knockout mice exhibit hearing loss, expansion of the inner ear compartments, and vestibular dysfunction (Hulander et al. 2003). Exonic *FOXI1* variants with conflicting pathogenicity classifications were detected in patients #307 and #678 (Additional file 2: Table S7). These were synonymous monoallelic variants and were therefore considered non-causative. Patient #307 also had biallelic pathogenic *GJB2* variants, which were most likely the cause of hearing loss. Patient #678 was submitted to ES but the cause of hearing loss remained undetermined (Table 3).

Homozygous or compound heterozygous pathogenic variants in the *KCNJ10* gene cause the SeSAMES syndrome, which features seizures, sensorineural deafness, ataxia, impaired intellectual development, and electrolyte imbalance (OMIM #612780). In our cohort, the maternally inherited monoallelic KCNJ10 variant p.R18Q was found in patient #616 (Additional file 2: Table S8) together with a monoallelic CEVA haplotype and the pathogenic monoallelic pendrin variant p.Y115D (Table 3). KCNJ10 variant p.R18Q was characterized as a gain-of-function by electrophysiology studies (Sicca et al. 2011). As hearing loss is associated with loss of function of the channel (Marcus et al. 2002; Freudenthal et al. 2011) it is unlikely that this variant contributed to the patient’s phenotype, which is most likely due to the association of the monoallelic CEVA haplotype and pendrin variant p.Y115D. Another monoallelic KCNJ10 variant with conflicting pathogenicity classifications (p.E177G) was found in patient #660 (Additional file 2: Table S8), together with biallelic pathogenic *SLC26A4* variants (Table 3). Although the contribution of the KCNJ10 variant is difficult to infer in this context, it is reasonable to assume that the phenotype of this patient was due to SLC26A4 dysfunction. Based on the above, *KCNJ10* and *FOXI1,* alone or in the context of digenic inheritance with *SLC26A4*, most likely did not contribute to hearing loss and EVA in our cohort.

Pathogenic sequence alterations in *GJB2* represent the leading cause of hereditary deafness in several world populations (Hilgert et al. 2009). Most pathogenic variants in this gene cause non-syndromic autosomal recessive hearing loss DFNB1, which is usually not associated with inner ear malformations (Kemperman et al. 2002). However, several studies reported monoallelic or biallelic *GJB2* variants in EVA patients (Lee et al. 2009; Propst et al. 2006; Schrijver and Chang 2006; Santos et al. 2010; Deklerck et al. 2015; Xiang et al. 2017; Wu et al. 2021). Evaluating whether findings of *GJB2* variants in EVA patients are causative or coincidental is challenging. *GJB2* and *SLC26A4* pathogenic variants can even be found in the same patient, *GJB2* causing hearing loss and *SLC26A4* causing EVA (Huang et al. 2013). In our EVA cohort, 2/32 (6%) patients (patients #307 and 421, Table 3 and Fig. 4) harbor biallelic pathogenic sequence alterations in *GJB2*. These patients are negative for all other known EVA genes tested (Additional file 2: Tables S6–S10), except for the monoallelic synonymous *FOXI1* variant in patient #307. Being most likely *GJB2* the gene causative for hearing loss, these patients were not submitted to ES. Thus, the genetic cause of EVA in these patients remains uncertain.

Digenic inheritance of non-syndromic hearing loss caused by *GJB2* and *GJB3* or *GJB6* has been described (del Castillo et al. 2005; Liu et al. 2009; Wilch et al. 2010). In our cohort, one patient (#669) harbors a monoallelic pathogenic sequence alteration in *GJB2*. No pathogenic sequence alterations in *GJB3* (Additional file 2: Table S9) or genomic deletions del(GJB6-D13S1830) and del(GJB6-D13S1854) at the *GJB6* locus were found in this patient and the entire cohort. Patient #669 was negative for all other genes tested and was submitted to ES but remained undiagnosed (Table 3).

*POU3F4* (OMIM *300039) encodes a transcription factor widely expressed in the neural tube during development (Mathis et al. 1992). Pathogenic sequence alterations in *POU3F4* lead to mixed conductive and sensorineural X-linked DFN3/DFNX2 deafness (OMIM #304400) associated with stapes fixation, cochlear incomplete partition type 3, and perilymphatic gusher during stapedectomy (de Kok et al. 1995). *POU3F4* sequence alterations do not invariably lead to EVA and, when present, EVA has specific anatomical features, is accompanied by other temporal bone deformities, and is seen in approximately 50% of cases. In our EVA cohort, pathogenic sequence alterations in *POU3F4* were found in 2/32 (6%) patients (Fig. 4), one with bilateral and one with unilateral EVA, and both with an IP3 (patients #569 and 667 respectively, Table 3). We have recently reported and characterized both variants as pathogenic (Bernardinelli et al. 2022). Of the 5/32 patients with unilateral EVA in our cohort, patient #667 was the only one who obtained a genetic diagnosis for his condition, pointing to the fact that sequencing the known EVA genes in the context of unilateral EVA will leave a significant proportion of cases unsolved, except in the presence of an IP3, which points to *POU3F4* as the causative factor.

Eleven carefully selected patients with bilateral EVA, bilateral hearing loss, and negative for known causative genes have been submitted to ES. ES detected 7 rare variants in 6 genes (*SCD5, REST, EDNRB, TJP2, TMC1*, and *CDH23*) formerly unrelated to EVA in 5/32 patients (16%, Tables 3 and 4 and Fig. 4). Referring only to the tested patients, ES detected putatively causative variants in 5/11 (45%) patients.

Patient #119 carries monoallelic non-synonymous variants within two genes, *REST* (OMIM ID: *600571) and *SCD5* (OMIM ID: *608370). *REST* encodes a transcriptional repressor. An intronic sequence alteration causing gain-of-function of REST in hair cells leads to autosomal dominant deafness 27 (DFNA27) (Nakano et al. 2018) while inactivating mutations lead to other forms of non-syndromic progressive autosomal dominant hearing loss (Manyisa et al. 2021) and Jones syndrome, a dominant syndrome characterized by gingival fibromatosis and progressive sensorineural hearing loss (Rahikkala et al. 2023). The *REST* variant *c.3272_3274delAAG* found in our patient leads to a single amino acid deletion at position 1091 in the 1097 amino acid protein product (p.E1091del) but preserves the integrity of the reading frame. *SCD5* encodes a stearoyl-CoA desaturase, an integral membrane protein of the endoplasmic reticulum that catalyzes the formation of monounsaturated fatty acids from saturated fatty acids. Pathogenic sequence alterations in *SCD5* cause autosomal dominant nonsyndromic progressive sensorineural hearing loss DFNA79 (Lu et al. 2020). The *SCD5 c.452T* > *G;* p.F151C variant found in our patient is a non-synonymous variant predicted to be damaging according to 5 prediction tools (Table 4). Both the variants identified in the patient within the *REST* and *SCD5* genes have never been characterized or directly associated with hearing loss and are classified as VUS according to the ACMG guidelines (Richards et al. 2015). Thus, considering that patient #119 has no family history of hearing loss and the genetic material of family members could not be obtained, it is currently unclear which of the two genes caused hearing loss and EVA in this patient.

*EDNRB* (OMIM ID: *131244) encodes for the non-selective endothelin receptor type B. Patient #359 carries the known pathogenic variant *c.757C* > *T* within *EDNRB* that creates a premature translational stop signal expected to result in an absent or disrupted protein product (p.R253*). This variant has been observed in individuals with clinical features of Waardenburg syndrome type 4A and segregated with the disease showing autosomal dominant inheritance (Syrris et al. 1999). The family originally described by Syrris et al. was of Afro-Caribbean origin and had variable manifestations of sensorineural deafness, heterochromia iridis, and Hirschsprung disease (aganglionic megacolon). Synophrys, hair or skin hypopigmentation, and dystopia canthorum were absent in this family. Patient #359 had sensorineural hearing loss with EVA and no overt signs of Waardenburg syndrome. Family history was positive for congenital deafness in the maternal aunt. However, the genetic material of the family members could not be obtained, and segregation studies could not be performed. EVA is found in approximately 50% of patients with Waardenburg syndrome types 1 and 2 (Watkinson et al. 2018), but is not typically reported in the context of Waardenburg syndrome type 4.

*TJP2* (OMIM ID: *607709) encodes the tight junction protein-2, or zonulin 2 (ZO-2), which belongs to a family of membrane-associated guanylate kinase (MAGUK) homologs involved in the organization of intercellular junctions by forming homo- and heterodimers with ZO-1 and ZO-3 and crosslinking transmembrane proteins, such as claudins and occludin, to the actin cytoskeleton (Itoh et al. 1999; Wu et al. 2007). The gene has been linked with autosomal recessive progressive familial intrahepatic cholestasis 4 and familial hypercholanemia 1. Both increased expression and decreased stability of TJP2 have been linked to autosomal dominant deafness 51 (DFNA51) (Walsh et al. 2010; Hilgert et al. 2008). A case of sporadic non-syndromic hearing loss with a homozygous *TJP2* variant has also been reported (Gu et al. 2015). Patient #568 carries the *c.1907C* > *G TJP2* variant leading to a dysfunctional protein product (p.T636R) in our cell-based assays, strongly supporting pathogenicity (Figs. 2 and 3). The TJP2 protein variant p.T636R failed to correctly localize to the cell periphery (Fig. 2) and remained trapped in the actin mesh (Fig. 3c, d) while conserving the ability to interact with the wild-type and affecting its cellular localization (Fig. 3a, b). This explains a monoallelic variant being sufficient to cause disease in this patient, consistent with an autosomal dominant pattern. This patient has no family history of hearing loss; therefore, either the mutation occurred de novo or hearing loss was incompletely penetrant in this family, as described for another *TJP2* variant (Rajabi et al. 2019). The genetic material of family members could not be obtained to discriminate between these two hypotheses.

*CDH23* (OMIM ID: *605516) is causative for autosomal recessive Usher syndrome type 1D (USH1D) and non-syndromic deafness DFNB12. While Usher syndrome is caused by homozygous or compound heterozygous nonsense, frameshift, splice site, and some missense mutations of *CDH23*, DFNB12 is associated with *CDH23* missense hypomorphic alleles with sufficient residual activity to preserve retinal and vestibular function, but not auditory function (Schultz et al. 2011). Of the two variants found in patient #632, *c.2289* + *1G* > *A* is a splicing variant established as pathogenic for Usher syndrome in an autosomal recessive manner. The second variant (*c.5677G* > *T*) is a missense variant classified as VUS, predicted to be damaging by five prediction tools (Table 4). The index patient has a sister with congenital deafness from whom the genetic material cannot be obtained. Therefore, segregation studies could not be performed in this family, and the phase (*cis* or *trans*) of the two variants detected in this patient is unknown. The index patient does not exhibit signs of Usher syndrome at 56 years of age. This can be explained by one DFNB12 allele *in trans* configuration to a USH1D allele of *CDH23* preserving vision and balance in deaf individuals (Schultz et al. 2011).

We explored a possible genotype–phenotype correlation in our cohort (Additional file 1: file S1), excluding patients with pathogenic biallelic *c.35delG GJB2* variants and *POU3F4* variants, which are known to lead to severe hearing loss phenotypes (Pollak et al. 2016; Cryns et al. 2004). The combination of an M1 genotype with the CEVA haplotype was reported to be associated with a less severe phenotype than the M2 genotype (Chao et al. 2019; Baldyga et al. 2023; Honda and Griffith 2022). In our five M2/M2 + CEVA patients, 9/10 ears had severe or profound hearing loss; in the group of four M1 + CEVA patients, 6/8 ears had severe or profound hearing loss. These differences did not reach statistical significance (*p* = 0.558). This is consistent with results from other groups (Smits et al. 2022). However, it should be noted that the patient with the mildest hearing loss phenotype (#616) belongs to the M1 + CEVA group. Also, there was no significant difference in hearing loss severity between M2/M2 + CEVA patients and M0 patients (excluding *GJB2* and *POU3F4* patients, 27/38 ears had a degree of hearing loss severe or higher; *p* = 0.413). Excluding from the M0 group those patients who have been diagnosed by ES, 17/28 ears had a degree of hearing loss severe or higher, which gave a *p* = 0.124 compared to the M2/M2 + CEVA patients. Compared to M2/M2 + CEVA patients, all of whom had bilateral EVA, undiagnosed patients with unilateral EVA had a significantly milder phenotype (only 3/8 ears had a degree of hearing loss severe or higher; *p* = 0.043). Undiagnosed patients with unilateral EVA had a significantly milder phenotype also compared to all patients with bilateral EVA (39/48 ears had a degree of hearing loss severe or higher, *p* = 0.018). This differs from previous reports (Archibald et al. 2019), and points to a different etiology of unilateral EVA in our cohort.

The detection of pathogenic variants in syndromic and/or dominant genes (*REST*, *SCD5*, *EDNRB*, and *TJP2*) in this cohort of patients with hearing loss and EVA is surprising, as no syndromic traits were observed at the clinical examination, and none of the natural parents of the index patients exhibited hearing loss. Hearing loss was occasionally present in other family members, but the unavailability of the genetic material impeded segregation studies and discrimination between de novo variants and incompletely penetrant phenotypes, which represent the main limitation of this study. Also, it must be considered that the variants in genes identified by ES (*REST*, *SCD5*, *EDNRB*, and *TJP2,* Table 4) can have been coincidentally detected in the context of an EVA due to other factors, such as unknown environmental factors or genetic factors not identified by ES. For example, deep intronic variants affecting the splicing, variants in regulatory regions, complex structural variants in known EVA genes or new candidate genes that cannot be detected by ES might have caused EVA in these patients. Whole Genome Sequencing can help verify this hypothesis. Concerning the variants detected in this study (Table 4), confirmatory studies in other EVA Caucasian cohorts, segregation studies, and mechanistic studies will be essential in discriminating between coincidental findings and causality.

## Conclusions

To conclude, in our central European Caucasian cohort with hearing loss and non-syndromic EVA, a combined genetic analysis approach prioritizing formerly known EVA-related genes detected biallelic pathogenic variants of *SLC26A4* in 5/32 patients (16%), monoallelic pathogenic *SLC26A4* variants with CEVA haplotype in 3/32 patients (9%), and a biallelic CEVA haplotype in 1/32 patients (3%). Functional and molecular tests have been instrumental in assigning or excluding the pathogenicity of *SLC26A4* variants. Pathogenic variants in *POU3F4* (2/32, 6%) and *GJB2* (2/32, 6%) were also found. CNV of *SLC26A4* and *STRC* or sequence alterations in *GJB3*, *GJB6*, *FOXI1*, and *KCNJ10* likely did not play a causative role. ES of undiagnosed patients with bilateral EVA detected rare sequence variants in 6 EVA-unrelated genes (*SCD5, REST, EDNRB, TJP2, TMC1*, and *CDH23*) in 5/11 patients (45%). Cell-based assays showed that the uncharacterized gene variant in *TJP2* leads to an aberrantly localized protein product conserving the ability of dimerization with the wild-type, supporting its autosomal dominant pathogenicity. The genetic causes of hearing loss and EVA remained unidentified in 44% (14/32) of patients, highlighting that pathogenic variants may lie in non-coding sequences of the human genome in a significant fraction of patients. Further studies are needed to demonstrate the pathomechanism of the CEVA haplotype and confirm the role of rare genes in other EVA cohorts.

## Supplementary Information

**Additional file 1.**

**Additional file 2.**

## Data Availability

The datasets used and/or analyzed during the current study are available from the corresponding author on reasonable request.

## Abbreviations

CEVA: Caucasian enlarged vestibular aqueduct
CNAs: Circulating nucleic acids
CT: Computed tomography
DFNA: Deafness, autosomal dominant
DFNB: Deafness, autosomal recessive
ECFP: Enhanced cyan fluorescent protein
EVA: Enlarged vestibular aqueduct
EYFP: Enhanced yellow fluorescent protein
FRET: Fluorescence resonance energy transfer
HEK: Human embryonic kidney
HL: Hearing loss
INDELS: Insertions/deletions
IP2/3: Incomplete cochlear partition type 2/3
MRI: Magnetic resonance imaging
SNP: Single nucleotide polymorphism
SNV: Single nucleotide variant
USH1D: Usher syndrome type 1D
VUS: Variant of uncertain significance
ES: Whole exome sequencing

## References

[CR1] Adzhubei I, Jordan DM, Sunyaev SR. Predicting functional effect of human missense mutations using PolyPhen-2. Curr Protoc Hum Genet. 2013;7:Unit7.20. 10.1002/0471142905.hg0720s76 PMC4480630 23315928

[CR2] Albert S, et al. SLC26A4 gene is frequently involved in nonsyndromic hearing impairment with enlarged vestibular aqueduct in Caucasian populations. Eur J Hum Genet. 2006;14:773–9. 16570074 10.1038/sj.ejhg.5201611

[CR3] Archibald HD, Ascha M, Gupta A, Megerian C, Otteson T. Hearing loss in unilateral and bilateral enlarged vestibular aqueduct syndrome. Int J Pediatr Otorhinolaryngol. 2019;118:147–51. 30634102 10.1016/j.ijporl.2018.12.023

[CR4] Baldyga N, et al. The genetic background of hearing loss in patients with EVA and cochlear malformation. Genes (Basel). 2023;14:335. 36833263 10.3390/genes14020335 PMC9957411

[CR5] Bernardinelli E, Costa R, Nofziger C, Paulmichl M, Dossena S. Effect of known inhibitors of ion transport on pendrin (SLC26A4) activity in a human kidney cell line. Cell Physiol Biochem. 2016;38:1984–98. 27161422 10.1159/000445559

[CR6] Bernardinelli E, et al. Novel POU3F4 variants identified in patients with inner ear malformations exhibit aberrant cellular distribution and lack of SLC6A20 transcriptional upregulation. Front Mol Neurosci. 2022;15: 999833. 36245926 10.3389/fnmol.2022.999833 PMC9558712

[CR7] Chao JR, et al. SLC26A4-linked CEVA haplotype correlates with phenotype in patients with enlargement of the vestibular aqueduct. BMC Med Genet. 2019;20:118. 31266487 10.1186/s12881-019-0853-4 PMC6604142

[CR8] Chattaraj P, et al. Use of SLC26A4 mutation testing for unilateral enlargement of the vestibular aqueduct. JAMA Otolaryngol Head Neck Surg. 2013;139:907–13. 24051746 10.1001/jamaoto.2013.4185

[CR9] Chattaraj P, et al. A common SLC26A4-linked haplotype underlying non-syndromic hearing loss with enlargement of the vestibular aqueduct. J Med Genet. 2017;54:665–73. 28780564 10.1136/jmedgenet-2017-104721 PMC5880640

[CR10] Chen S, et al. A genomic mutational constraint map using variation in 76,156 human genomes. Nature. 2024;625:92–100. 38057664 10.1038/s41586-023-06045-0 PMC11629659

[CR11] Choe G, Park SK, Kim BJ. Hearing loss in neonates and infants. Clin Exp Pediatr. 2023;66:369–76. 36634668 10.3345/cep.2022.01011 PMC10475863

[CR12] Choi BY, et al. Hypo-functional SLC26A4 variants associated with nonsyndromic hearing loss and enlargement of the vestibular aqueduct: genotype–phenotype correlation or coincidental polymorphisms? Hum Mutat. 2009;30:599–608. 19204907 10.1002/humu.20884 PMC2663020

[CR13] Cirello V, et al. Molecular and functional studies of 4 candidate loci in Pendred syndrome and nonsyndromic hearing loss. Mol Cell Endocrinol. 2012;351:342–50. 22285650 10.1016/j.mce.2012.01.013

[CR14] Cryns K, et al. A genotype–phenotype correlation for GJB2 (connexin 26) deafness. J Med Genet. 2004;41:147–54. 14985372 10.1136/jmg.2003.013896 PMC1735685

[CR15] de Kok YJ, et al. Association between X-linked mixed deafness and mutations in the POU domain gene POU3F4. Science. 1995;267:685–8. 7839145 10.1126/science.7839145

[CR16] de Moraes VCS, et al. Reduction of cellular expression levels is a common feature of functionally affected pendrin (SLC26A4) protein variants. Mol Med. 2016;22:41–53. 26752218 10.2119/molmed.2015.00226 PMC5004711

[CR17] Deklerck AN, Acke FR, Janssens S, De Leenheer EM. Etiological approach in patients with unidentified hearing loss. Int J Pediatr Otorhinolaryngol. 2015;79:216–22. 25555640 10.1016/j.ijporl.2014.12.012

[CR18] del Castillo FJ, et al. A novel deletion involving the connexin-30 gene, del(GJB6-d13s1854), found in trans with mutations in the GJB2 gene (connexin-26) in subjects with DFNB1 non-syndromic hearing impairment. J Med Genet. 2005;42:588–94. 15994881 10.1136/jmg.2004.028324 PMC1736094

[CR19] Denoyelle F, et al. Prelingual deafness: high prevalence of a 30delG mutation in the connexin 26 gene. Hum Mol Genet. 1997;6:2173–7. 9336442 10.1093/hmg/6.12.2173

[CR20] Dossena S, et al. Fast fluorometric method for measuring pendrin (SLC26A4) Cl-/I-transport activity. Cell Physiol Biochem. 2006;18:67–74. 16914891 10.1159/000095164

[CR21] Dossena S, et al. Identification of allelic variants of pendrin (SLC26A4) with loss and gain of function. Cell Physiol Biochem. 2011a;28:467–76. 22116359 10.1159/000335108 PMC3709191

[CR22] Dossena S, et al. Functional characterization of pendrin mutations found in the Israeli and Palestinian populations. Cell Physiol Biochem. 2011b;28:477–84. 22116360 10.1159/000335109 PMC3709187

[CR23] Dossena S, et al. Molecular and functional characterization of human pendrin and its allelic variants. Cell Physiol Biochem. 2011c;28:451–66. 22116358 10.1159/000335107

[CR24] Dror AA, et al. Calcium oxalate stone formation in the inner ear as a result of an Slc26a4 mutation. J Biol Chem. 2010;285:21724–35. 20442411 10.1074/jbc.M110.120188 PMC2898392

[CR25] Enerback S, et al. Acidosis and deafness in patients with recessive mutations in FOXI1. J Am Soc Nephrol. 2018;29:1041–8. 29242249 10.1681/ASN.2017080840 PMC5827603

[CR26] Everett LA, et al. Pendred syndrome is caused by mutations in a putative sulphate transporter gene (PDS). Nat Genet. 1997;17:411–22. 9398842 10.1038/ng1297-411

[CR27] Freudenthal B, et al. KCNJ10 mutations disrupt function in patients with EAST syndrome. Nephron Physiol. 2011;119:40–8. 10.1159/000330250 21849804

[CR28] Frohne A, et al. Mutational spectrum in patients with dominant non-syndromic hearing loss in Austria. Eur Arch Otorhinolaryngol. 2024;281:3577–86. 38400873 10.1007/s00405-024-08492-5 PMC11211180

[CR29] Fugazzola L, Cerutti N, Mannavola D, Vannucchi G, Beck-Peccoz P. The role of pendrin in iodide regulation. Exp Clin Endocrinol Diabetes. 2001;109:18–22. 11573133 10.1055/s-2001-11008

[CR30] Fugazzola L, et al. High phenotypic intrafamilial variability in patients with Pendred syndrome and a novel duplication in the SLC26A4 gene: clinical characterization and functional studies of the mutated SLC26A4 protein. Eur J Endocrinol. 2007;157:331–8. 17766716 10.1530/EJE-07-0263

[CR31] Galietta LJ, Haggie PM, Verkman AS. Green fluorescent protein-based halide indicators with improved chloride and iodide affinities. FEBS Lett. 2001;499:220–4. 11423120 10.1016/s0014-5793(01)02561-3

[CR32] Gillam MP, Bartolone L, Kopp P, Bevenga S. Molecular analysis of the PDS gene in a nonconsanguineous Sicilian family with Pendred’s syndrome. Thyroid. 2005;15:734–41. 16053392 10.1089/thy.2005.734

[CR33] Gong WX, Gong RZ, Zhao B. HRCT and MRI findings in X-linked non-syndromic deafness patients with a POU3F4 mutation. Int J Pediatr Otorhinolaryngol. 2014;78:1756–62. 25175280 10.1016/j.ijporl.2014.08.013

[CR34] Griffith AJ, Wangemann P. Hearing loss associated with enlargement of the vestibular aqueduct: mechanistic insights from clinical phenotypes, genotypes, and mouse models. Hear Res. 2011;281:11–7. 21669267 10.1016/j.heares.2011.05.009 PMC3183377

[CR35] Gu X, et al. Genetic testing for sporadic hearing loss using targeted massively parallel sequencing identifies 10 novel mutations. Clin Genet. 2015;87:588–93. 24853665 10.1111/cge.12431

[CR36] Hereditary Hearing Loss Homepage (Internet). Available from: https://hereditaryhearingloss.org. Accessed on 01.06.2024.

[CR37] Hilgert N, et al. Mutation analysis of TMC1 identifies four new mutations and suggests an additional deafness gene at loci DFNA36 and DFNB7/11. Clin Genet. 2008;74:223–32. 18616530 10.1111/j.1399-0004.2008.01053.x PMC4732719

[CR38] Hilgert N, Smith RJ, Van Camp G. Forty-six genes causing nonsyndromic hearing impairment: Which ones should be analyzed in DNA diagnostics? Mutat Res. 2009;681:189–96. 18804553 10.1016/j.mrrev.2008.08.002 PMC2847850

[CR39] Honda K, Griffith AJ. Genetic architecture and phenotypic landscape of SLC26A4-related hearing loss. Hum Genet. 2022;141:455–64. 34345941 10.1007/s00439-021-02311-1

[CR40] Huang S, et al. Sensorineural hearing loss caused by mutations in two alleles of both GJB2 and SLC26A4 genes. Int J Pediatr Otorhinolaryngol. 2013;77:379–83. 23266159 10.1016/j.ijporl.2012.11.031

[CR41] Hulander M, et al. Lack of pendrin expression leads to deafness and expansion of the endolymphatic compartment in inner ears of Foxi1 null mutant mice. Development. 2003;130:2013–25. 12642503 10.1242/dev.00376

[CR42] Ito T, et al. SLC26A4 mutation testing for hearing loss associated with enlargement of the vestibular aqueduct. World J Otorhinolaryngol. 2013;3:26–34. 25960948 10.5319/wjo.v3.i2.26 PMC4423814

[CR43] Itoh M, et al. Direct binding of three tight junction-associated MAGUKs, ZO-1, ZO-2, and ZO-3, with the COOH termini of claudins. J Cell Biol. 1999;147:1351–63. 10601346 10.1083/jcb.147.6.1351 PMC2168087

[CR44] Jonard L, et al. Genetic evaluation of prelingual hearing impairment: recommendations of an European network for genetic hearing impairment. Audiol Res. 2023;13:341–6. 37218840 10.3390/audiolres13030029 PMC10204519

[CR45] Kemperman MH, Hoefsloot LH, Cremers CW. Hearing loss and connexin 26. J R Soc Med. 2002;95:171–7. 11934905 10.1258/jrsm.95.4.171 PMC1279509

[CR46] Kenna MA, Rehm HL, Frangulov A, Feldman HA, Robson CD. Temporal bone abnormalities in children with GJB2 mutations. Laryngoscope. 2011;121:630–5. 21298644 10.1002/lary.21414 PMC3061391

[CR47] Koffler T, Ushakov K, Avraham KB. Genetics of hearing loss: syndromic. Otolaryngol Clin North Am. 2015;48:1041–61. 26443487 10.1016/j.otc.2015.07.007 PMC4641804

[CR48] Landa P, Differ AM, Rajput K, Jenkins L, Bitner-Glindzicz M. Lack of significant association between mutations of KCNJ10 or FOXI1 and SLC26A4 mutations in Pendred syndrome/enlarged vestibular aqueducts. BMC Med Genet. 2013;14:85. 23965030 10.1186/1471-2350-14-85 PMC3765178

[CR49] Lee KH, et al. Audiologic and temporal bone imaging findings in patients with sensorineural hearing loss and GJB2 mutations. Laryngoscope. 2009;119:554–8. 19235794 10.1002/lary.20162 PMC7065710

[CR50] Leung KJ, Quesnel AM, Juliano AF, Curtin HD. Correlation of CT, MR, and histopathology in incomplete partition-II cochlear anomaly. Otol Neurotol. 2016;37:434–7. 27093025 10.1097/MAO.0000000000001027

[CR51] Limongelli I, Marini S, Bellazzi R. PaPI: pseudo amino acid composition to score human protein-coding variants. BMC Bioinform. 2015;16:123. 10.1186/s12859-015-0554-8 PMC4411653 25928477

[CR52] Liu XZ, et al. Digenic inheritance of non-syndromic deafness caused by mutations at the gap junction proteins Cx26 and Cx31. Hum Genet. 2009;125:53–62. 19050930 10.1007/s00439-008-0602-9 PMC2737700

[CR53] Livak KJ, Schmittgen TD. Analysis of relative gene expression data using real-time quantitative PCR and the 2(− Delta Delta C(T)) method. Methods. 2001;25:402–8. 11846609 10.1006/meth.2001.1262

[CR54] Lu X, et al. Whole exome sequencing identifies SCD5 as a novel causative gene for autosomal dominant nonsyndromic deafness. Eur J Med Genet. 2020;63: 103855. 31972369 10.1016/j.ejmg.2020.103855

[CR55] Makretskaya N, et al. High frequency of mutations in ‘dyshormonogenesis genes’ in severe congenital hypothyroidism. PLoS ONE. 2018;13: e0204323. 30240412 10.1371/journal.pone.0204323 PMC6150524

[CR56] Manyisa N, et al. A monoallelic variant in REST is associated with non-syndromic autosomal dominant hearing impairment in a South African family. Genes (Basel). 2021;12:1765. 34828371 10.3390/genes12111765 PMC8618167

[CR57] Marcus DC, Wu T, Wangemann P, Kofuji P. KCNJ10 (Kir4.1) potassium channel knockout abolishes endocochlear potential. Am J Physiol Cell Physiol. 2002;282:C403-407. 11788352 10.1152/ajpcell.00312.2001

[CR58] Mathis JM, Simmons DM, He X, Swanson LW, Rosenfeld MG. Brain 4: a novel mammalian POU domain transcription factor exhibiting restricted brain-specific expression. The EMBO J. 1992;11:2551–61. 1628619 10.1002/j.1460-2075.1992.tb05320.x PMC556730

[CR59] Matulevicius A, et al. Molecular features of SLC26A4 common variant p.L117F. J Clin Med. 2022;11:5549. 36233414 10.3390/jcm11195549 PMC9570580

[CR60] Nakano Y, et al. Defects in the alternative splicing-dependent regulation of REST cause deafness. Cell. 2018;174(536–548): e521. 10.1016/j.cell.2018.06.004 PMC6370011 29961578

[CR61] Ng PC, Henikoff S. SIFT: predicting amino acid changes that affect protein function. Nucl Acids Res. 2003;31:3812–4. 12824425 10.1093/nar/gkg509 PMC168916

[CR62] Pandya A. Genetic hearing loss: the journey of discovery to destination—How close are we to therapy? Mol Genet Genomic Med. 2016;4:583–7. 27896280 10.1002/mgg3.260 PMC5118202

[CR63] Pera A, et al. Functional assessment of allelic variants in the SLC26A4 gene involved in Pendred syndrome and nonsyndromic EVA. Proc Natl Acad Sci USA. 2008a;105:18608–13. 19017801 10.1073/pnas.0805831105 PMC2584577

[CR64] Pera A, et al. A mutational analysis of the SLC26A4 gene in Spanish hearing-impaired families provides new insights into the genetic causes of Pendred syndrome and DFNB4 hearing loss. Eur J Hum Genet. 2008b;16:888–96. 18285825 10.1038/ejhg.2008.30

[CR65] Pique LM, et al. Mutation analysis of the SLC26A4, FOXI1 and KCNJ10 genes in individuals with congenital hearing loss. PeerJ. 2014;2: e384. 24860705 10.7717/peerj.384 PMC4017815

[CR66] Pollak A, et al. Novel and de novo mutations extend association of POU3F4 with distinct clinical and radiological phenotype of hearing loss. PLoS ONE. 2016;11: e0166618. 27941975 10.1371/journal.pone.0166618 PMC5152817

[CR67] Pos O, Biro O, Szemes T, Nagy B. Circulating cell-free nucleic acids: characteristics and applications. Eur J Hum Genet. 2018;26:937–45. 29681621 10.1038/s41431-018-0132-4 PMC6018748

[CR68] Procino G, et al. Co-regulated pendrin and aquaporin 5 expression and trafficking in Type-B intercalated cells under potassium depletion. Cell Physiol Biochem. 2013;32:184–99. 24429825 10.1159/000356638

[CR69] Propst EJ, et al. Temporal bone imaging in GJB2 deafness. Laryngoscope. 2006;116:2178–86. 17146393 10.1097/01.mlg.0000244389.68568.a7

[CR70] Quaio C, et al. Genomic study of nonsyndromic hearing loss in unaffected individuals: frequency of pathogenic and likely pathogenic variants in a Brazilian cohort of 2097 genomes. Front Genet. 2022;13: 921324. 36147510 10.3389/fgene.2022.921324 PMC9486813

[CR71] Quang D, Chen Y, Xie X. DANN: a deep learning approach for annotating the pathogenicity of genetic variants. Bioinformatics. 2015;31:761–3. 25338716 10.1093/bioinformatics/btu703 PMC4341060

[CR72] Rahikkala E, et al. Pathogenic REST variant causing Jones syndrome and a review of the literature. Eur J Hum Genet. 2023;31:469–73. 36509837 10.1038/s41431-022-01258-9 PMC10133349

[CR73] Rajabi S, et al. TJP2 gene mutation c.G1012A may be responsible for congenital hearing loss with incomplete penetrance in an Iranian Pedigree. J Genet Resour. 2019;5:143–8.

[CR74] Richards S, et al. Standards and guidelines for the interpretation of sequence variants: a joint consensus recommendation of the American College of Medical Genetics and Genomics and the Association for Molecular Pathology. Genet Med. 2015;17:405–24. 25741868 10.1038/gim.2015.30 PMC4544753

[CR75] Roesch S, et al. Functional testing of SLC26A4 variants-clinical and molecular analysis of a cohort with enlarged vestibular aqueduct from Austria. Int J Mol Sci. 2018;19:209. 29320412 10.3390/ijms19010209 PMC5796158

[CR76] Roesch S, Rasp G, Sarikas A, Dossena S. Genetic determinants of non-syndromic enlarged vestibular aqueduct: a review. Audiol Res. 2021;11:423–42. 34562878 10.3390/audiolres11030040 PMC8482117

[CR77] Santos S, et al. Enlarged vestibular aqueduct syndrome. A review of 55 paediatric patients. Acta Otorrinolaringol Esp. 2010;61:338–44. 20684821 10.1016/j.otorri.2010.04.002

[CR78] Schrijver I, Chang KW. Two patients with the V37I/235delC genotype: are radiographic cochlear anomalies part of the phenotype? Int J Pediatr Otorhinolaryngol. 2006;79:216–22. 10.1016/j.ijporl.2006.07.015 16952406

[CR79] Schultz JM, et al. Allelic hierarchy of CDH23 mutations causing non-syndromic deafness DFNB12 or Usher syndrome USH1D in compound heterozygotes. J Med Genet. 2011;48:767–75. 21940737 10.1136/jmedgenet-2011-100262

[CR80] Seiler CY, et al. DNASU plasmid and PSI:biology-materials repositories: resources to accelerate biological research. Nucl Acids Res. 2014;42:D1253-1260. 24225319 10.1093/nar/gkt1060 PMC3964992

[CR81] Sennaroglu L, Saatci I. A new classification for cochleovestibular malformations. Laryngoscope. 2002;112:2230–41. 12461346 10.1097/00005537-200212000-00019

[CR82] Sicca F, et al. Autism with seizures and intellectual disability: possible causative role of gain-of-function of the inwardly-rectifying K+ channel Kir4.1. Neurobiol Dis. 2011;43:239–47. 21458570 10.1016/j.nbd.2011.03.016

[CR83] Smith RJH, Iwasa Y, Schaefer AM, et al. Pendred syndrome/nonsyndromic enlarged vestibular aqueduct. In: Adam MP, et al., editors. GeneReviews((R)). Seattle: Springer; 1993.

[CR84] Smits JJ, et al. Exploring the missing heritability in subjects with hearing loss, enlarged vestibular aqueducts, and a single or no pathogenic SLC26A4 variant. Hum Genet. 2022;141:465–84. 34410491 10.1007/s00439-021-02336-6 PMC9035008

[CR85] Syrris P, Carter ND, Patton MA. Novel nonsense mutation of the endothelin-B receptor gene in a family with Waardenburg–Hirschsprung disease. Am J Med Genet. 1999;87:69–71. 10528251

[CR86] Usami S, et al. Non-syndromic hearing loss associated with enlarged vestibular aqueduct is caused by PDS mutations. Hum Genet. 1999;104:188–92. 10190331 10.1007/s004390050933

[CR87] Vijayasekaran S, et al. When is the vestibular aqueduct enlarged? A statistical analysis of the normative distribution of vestibular aqueduct size. AJNR Am J Neuroradiol. 2007;28:1133–8. 17569973 10.3174/ajnr.A0495 PMC8134171

[CR88] Walsh T, et al. Genomic duplication and overexpression of TJP2/ZO-2 leads to altered expression of apoptosis genes in progressive nonsyndromic hearing loss DFNA51. Am J Hum Genet. 2010;87:101–9. 20602916 10.1016/j.ajhg.2010.05.011 PMC2896780

[CR89] Wasano K, et al. Systematic quantification of the anion transport function of pendrin (SLC26A4) and its disease-associated variants. Hum Mutat. 2020;41:316–31. 31599023 10.1002/humu.23930 PMC6930342

[CR90] Watkinson JC, et al. Balance disorders in children. In: Watkinson J, Clarke R, editors., et al., Scott–Brown’s otorhinolaryngology and head and neck surgery. London: CRC Press; 2018.

[CR91] Wilch E, et al. A novel DFNB1 deletion allele supports the existence of a distant cis-regulatory region that controls GJB2 and GJB6 expression. Clin Genet. 2010;78:267–74. 20236118 10.1111/j.1399-0004.2010.01387.x PMC2919588

[CR92] Wu J, et al. Domain-swapped dimerization of the second PDZ domain of ZO2 may provide a structural basis for the polymerization of claudins. J Biol Chem. 2007;282:35988–99. 17897942 10.1074/jbc.M703826200

[CR93] Wu H, et al. Validation and analysis of Goldengate high-throughput deafness gene chip in detecting the patients with enlarged vestibular aqueduct syndrome. Zhonghua Er Bi Yan Hou Tou Jing Wai Ke Za Zhi. 2021;56:138–43. 33548943 10.3760/cma.j.cn115330-20200302-00150

[CR94] Xiang Y, et al. Mutation analysis and prenatal diagnosis for 12 families affected with hereditary hearing loss and enlarged vestibular aqueduct. Zhonghua Yi Xue Yi Chuan Xue Za Zhi. 2017;34:336–41. 28604950 10.3760/cma.j.issn.1003-9406.2017.03.005

[CR95] Yang T, et al. Transcriptional control of SLC26A4 is involved in Pendred syndrome and nonsyndromic enlargement of vestibular aqueduct (DFNB4). Am J Hum Genet. 2007;80:1055–63. 17503324 10.1086/518314 PMC1867094

[CR96] Yang T, et al. Mutations of KCNJ10 together with mutations of SLC26A4 cause digenic nonsyndromic hearing loss associated with enlarged vestibular aqueduct syndrome. Am J Hum Genet. 2009;84:651–7. 19426954 10.1016/j.ajhg.2009.04.014 PMC2681005

[CR97] Yuan Y, et al. Molecular epidemiology and functional assessment of novel allelic variants of SLC26A4 in non-syndromic hearing loss patients with enlarged vestibular aqueduct in China. PLoS ONE. 2012;7: e49984. 23185506 10.1371/journal.pone.0049984 PMC3503781

