# Supplementary material for: Novel genetic determinants contribute to hearing loss in a central European cohort with enlarged vestibular aqueduct
Source: Mol Med. 2025 Mar 22;31:111. doi: 10.1186/s10020-025-01159-9 (PMC11929268; doi:10.1186/s10020-025-01159-9)
Supplement: Supplementary file 2 — Additional file 2. [file 10020_2025_1159_MOESM2_ESM.docx]

**Novel genetic determinants contribute to hearing loss in a central European cohort with enlarged Vestibular Aqueduct**

**Emanuele Bernardinelli, Raffaella Liuni, Rapolas Jamontas, Paola Tesolin, Anna Morgan,**

**Giorgia Girotto, Sebastian Roesch, Silvia Dossena**

**SUPPLEMENTAL INFORMATION**

**Supplemental methods**

***Characterization of hearing loss***

Characterization of hearing loss (HL) was based on individual side-specific pure-tone audiometric testing. The type of HL was defined as conductive, mixed, or sensorineural based on air- and bone-conduction thresholds. The severity of HL was classified on decibel hearing level (dB HL) thresholds, either as mild for values between 26 and 40 dB HL, moderate between 41 and 60 dB HL, severe between 61 and 80 dB HL, or profound for values above 80 dB HL. Ears with no perception were defined as deaf. Frequencies affected by HL were divided into low, middle, or high-frequency. The onset of HL was defined through the patient’s history. Related to the time of speech development, the onset of HL was defined as congenital, prelingual, perilingual, or postlingual. The clinical course of HL with possible hearing drops was obtained through audiometric tests and the patient’s history. Surgical reports were screened for intraoperative gusher phenomenon, defined as the visible efflux of cerebrospinal fluid after cochleostomy during cochlear implant surgery.

***Cell culture***

Human Embryonic Kidney (HEK) 293 Phoenix (1) and HeLa cells (human cervical adenocarcinoma, CCL-2, directly obtained from American Type Cell Culture Collection (ATCC, Manassas, VA, United States) were cultured in Minimum Essential Eagle Medium (Sigma-Aldrich, St. Louis, MO, United States) supplemented with 10% fetal bovine serum (GIBCO, Thermo Fisher Scientific, Waltham, MA, United States), 2 mM L-glutamine, 100 U/ml penicillin, 100 mg/ml streptomycin, and 1 mM pyruvic acid (sodium salt). The cells were maintained at 37 °C, 5% CO_2_, 95% air, and 100% humidity. Subcultures were routinely established every second to third day by seeding the cells into 100-mm diameter Petri dishes following trypsin/ethylenediaminetetraacetic acid (EDTA) treatment.

***SLC26A4 ion transport measurements***

The ion transport function of SLC26A4 was measured via a fluorometric method allowing for evaluation of the iodide influx in SLC26A4-transfected cells. Cells were initially washed and bathed in 70 μl of a high-chloride solution (in mM: KCl 2, NaCl 135, CaCl_2_ 1, MgCl_2_ 1, D-glucose 10, 4-(2-hydroxyethyl)-1-piperazineethane sulfonic acid (HEPES) 20, 308 mOsm/KgH_2_O adjusted with mannitol, pH 7.4), and the baseline fluorescence intensity was measured (1 measurement/sec for 3 sec). Subsequently, 140 μl of a high-iodide solution (in mM: KCl 2, NaI 135, CaCl_2_ 1, MgCl_2_ 1, D-glucose 10, HEPES 20, 308 mOsm/KgH_2_O adjusted with mannitol, pH 7.4) were injected into each well and the fluorescence intensity was measured for 16 sec. Fluorescence intensity was quantified with the VICTOR^TM^ X3 Multilabel Plate Reader (Perkin Elmer, Waltham, MA, USA) equipped with a liquid dispenser and the following filters: excitation: F485 (excitation center wavelength (CWL): 485 nm, bandwidth: 14 nm), emission: F535 (emission CWL: 535 nm, bandwidth: 25 nm). The background fluorescence was subtracted from all of the other fluorescence measurements of the same 96-well plate. Data are expressed as % fluorescence variations and a negative fluorescence variation indicates a flux of iodide from the extracellular milieu to the intracellular environment. Experiments were performed at room temperature.

***Determination of SLC26A4 protein expression levels by quantitative imaging***

For quantitative imaging, EYFP was excited with the 514 nm line of the Argon laser and emission was detected between 525 and 600 nm; DAPI was excited with a diode laser (405 nm) and emission was detected between 430 and 470 nm. Laser power and photomultipliers gain were kept rigorously constant for the acquisition of all images. To normalize SLC26A4 expression levels for the cell density, the EYFP fluorescence intensity of whole imaging fields was expressed as average levels of gray, subtracted for the background fluorescence, and normalized for the background-subtracted fluorescence intensity of DAPI.

***Colocalization***

The impact of TJP2 variant p.T636R on subcellular localization was determined in living HEK 293 Phoenix cells by co-localization of wild-type or mutant TJP2-EYFP and the plasma membrane. To stain the plasma membrane, cells were incubated on ice with 1.25 μg/ml CellMask^TM^ Deep Red Plasma Membrane Stain (C10046, Invitrogen Molecular Probes, Waltham, MA, USA) in HBSS for 5 min and washed in ice-cold HBSS. Imaging was performed by sequential acquisition with a Leica TCS SP5II AOBS confocal microscope 30 hours after transfection. EYFP was excited with the 514 nm line of the Argon laser, and emission was detected in the 525–600 nm range; the CellMask^TM^ Deep Red Plasma Membrane Stain was excited at 633 nm (HeNe laser), and emission was detected in the 650–750 nm range. Co-localization was quantified in single cells with the Colocalization tool of the LAS AF SP5 software (Leica Microsystems) and represented as Pearson's correlation coefficient (2), overlap coefficient, and co-localization rate.

***Fluorescence Resonance Energy Transfer***

EYFP was excited with the 514-nm line of the Argon laser, and the emission was detected in the 525–600 nm range. ECFP was excited with the 405 nm laser, and the emission was detected in the 445–490 nm range. EYFP photobleaching in whole cells was obtained with 15 sequential illuminations at 514 nm (zoom factor 8×). FRET efficiency was calculated using the formula:

FRET efficiency = 1−ECFPpre/ECFPpost

where ECFPpre and ECFPpost refer to the average ECFP intensity before and after EYFP photobleaching, respectively, in a region of interest corresponding to an individual cell within the bleaching region.

**Table S1. Target amplification and Sanger sequencing primers.**

| **Gene** | **Amplification** | **Sequencing** |
| --- | --- | --- |
| **CEVA SNP rs199915614** | Sense, 5’ CCGTTTACAACAGACATTTCAGAAGAG 3’  Antisense, 5’ GTAGCTGCCTTCAGAATGCCAC 3’ | Sense, 5’ CCGTTTACAACAGACATTTCAGAAGAG 3’  Antisense, 5’ GTAGCTGCCTTCAGAATGCCAC 3’ |
| ***FOXI1*** | Sense, 5’ TAGCATGTCATTAGTGGG 3’  Antisense, 5´AACAGAAGCCTTAACCAG 3´ | Sense, 5’ TAGCATGTCATTAGTGGG 3’  Antisense, 5’ GCTGAAACCCAAATCTTC 3’  Sense, 5´CCTCCATTTCTCAGTCCC 3´  Antisense, 5´AACAGAAGCCTTAACCAG 3´ |
| ***GJB2*** | As in (3) | |
| ***GJB3*** | Sense, 5’ CAGCTTGGGAGGAATAACAGTTC 3’  Antisense, 5’ GTTAATTCGCTTGTTTCAAAGTCTATG 3’ | Sense, 5’ CAGCTTGGGAGGAATAACAGTTC 3’  Antisense, 5’ GTTAATTCGCTTGTTTCAAAGTCTATG 3’  Sense, 5’ CTACCTGTTCAGCCTCATCTTC 3’  Antisense, 5’ GATTTTCTTCTCGGTAGGTCGG 3’ |
| ***KCNJ10*** | Sense, 5´ AGTAGCTGGGACTACAGGCGCA 3´  Antisense, 5´AAAGGGGACAGTGGGAGGCGAA 3´ | Sense, 5’ TAATTCCTCCCTCCCATG 3’  Antisense, 5’ CAGGGCATTGGAAGAGAG 3’  Sense, 5´ GATCCGAGTTGCCAATATGC 3’  Antisense, 5´ CACATTGACCTGGTTGAGCC 3’ |
| ***POU3F4*** | Sense, 5' TATTGGCCGGGCTTACTCC 3'  Antisense, 5' AGATCCCAGCTTGGACTGC 3' | Sense, 5' TATTGGCCGGGCTTACTCC 3'  Antisense, 5' AGATCCCAGCTTGGACTGC 3'  Sense, 5’ AACGTGTACTCGCAGCCTG 3’  Antisense, 5’ GTCTCCTCGTCGGAGTGATC 3’ |
| ***SLC26A4*** | As in (3) | |

**Table S2. Mutagenesis primers.**

| **Variant** | **Sense** | **Antisense** |
| --- | --- | --- |
| **SLC26A4 p.Y78C** | 5’- GTGGCTCCCCAAATGCCGAGTCAAGGAATG -3’ | 5’- CATTCCTTGACTCGGCATTTGGGGAGCCAC-3’ |
| **SLC26A4 p.Q101R** | 5'- CTAGTGGCCACGCTGCGAGGGATGGCATATG -3' | 5'- CATATGCCATCCCTCGCAGCGTGGCCACTAG -3' |
| **SLC26A4 p.I136N** | 5'- CATCAAGACATAACTCAGTTGGACCTTTTC -3' | 5'- GAAAAGGTCCAACTGAGTTATGTCTTGATG -3' |
| **SLC26A4 p.L597S** | 5'- CTAATAAAAAGTGGACAATCAAGAGCAACAAAG -3' | 5'- CTTTGTTGCTCTTGATTGTCCACTTTTTATTAG -3' |
| **SLC26A4 p.A664V** | 5'- CTGCTTGACTGTGGAGTTATATCTTTCCTG -3' | 5'- CAGGAAAGATATAACTCCACAGTCAAGCAG -3' |
| **SLC26A4 p.G740V** | 5'- CTCAAGAGGGTCAAGTTTCCATTTTAGAAACG -3' | 5'- CGTTTCTAAAATGGAAACTTGACCCTCTTGAG-3' |
| **TJP2 p.T636R** | 5’- GAGTGGTAGACAGACTGTATGACGGCAAG -3’ | 5’- CTTGCCGTCATACAGTCTGTCTACCACTC -3’ |

**Table S3: Additional clinical features of the newly recruited patients concerning the hearing phenotype.** B, bilateral, HL, hearing loss, L, left, R, right. Age is given in years unless otherwise specified.

| **Patient** | **Patient ID** | **Severity of HL,**  **Side** | **Onset of HL (years)** | **Clinical**  **course of HL, Side** | **Hearing**  **drops** | **Cochlear Implant,**  **Side, Age at implantation** | **Intra-operative Gusher** | **Family history of HL** |
| --- | --- | --- | --- | --- | --- | --- | --- | --- |
| **17** | **#653** | Profound | Congenital | L stable,  R progressive | No | L, age 4 | Yes | None |
| **18** | **#654** | Profound | Congenital | Stable | No | B, age 1 | Yes | Yes, unilateral HL in a maternal aunt |
| **19** | **#657** | Profound | Postlingual | Fluctuating | Yes | No | - | None |
| **20** | **#659** | Profound | Unclear, diagnosis at the age of 3 | R stable,  L progressive | R no,  L yes | R, age 24 | Unknown | None |
| **21** | **#660** | Profound | Congenital | Stable | No | R, age 21 | No | Yes, HL in one of two sisters |
| **22** | **#663** | R profound, L mild | Postlingual, diagnosis at the age of 6 | Stable | No | R, age 38 | No | Yes, HL in the brother; nephew with hearing drops |
| **23** | **#666** | B mild, conductive only | Diagnosis at the age of 2 | Stable | No | No | - | None |
| **24** | **#667** | Profound | Prelingual, diagnosis at the age of 9 months | R progressive,  L stable | No | R, age 8 | Yes | None |
| **25** | **#669** | Profound | Postlingual | Progressive | Yes | L, age 51 | No | None |
| **26** | **#670** | Profound | Congenital | Stable | No | B, age 2 | Yes | None |
| **27** | **#671** | R profound,  L profound for frequencies >1 kHz | Postlingual; R after infection at age of 7;  L hearing drops from age of 29 | R stable,  L fluctuating | Yes | R, age 46 | Yes | Yes; 1 sister congenitally deaf |
| **28** | **#672** | R mild,  L profound | Congenital | Stable | No | No | - | None |
| **29** | **#678** | Profound | Congenital | Stable | No | R age 14 , L age 36 | No | None |
| **30** | **#679** | Profound | Postlingual, diagnosis at the age of 16 | Progressive | R yes, age 16 | L, age 62 | No | None |
| **31** | **#680** | R profound | Postlingual, onset during childhood | Stable | No | R, age 51 | Yes | Unknown |
| **32** | **#681** | R profound,  L severe | Prelingual,  diagnosis at age of 2 | Progressive | Unknown | R, age 3 | Unknown | None |

**Table S4: Clinical features of the newly recruited patients concerning the vestibular function.** n/a, not assessed. aSPV, average slow phase velocity of nystagmus, degrees/second.

| **Patient** | **Patient ID** | **Subjective Vertigo** | **Problems in learning to walk** | **Vestibular function** | | **Migraine** |
| --- | --- | --- | --- | --- | --- | --- |
|  |  |  |  | **Caloric testing - slow phase velocity of nystagmus** | **Video Head Impulse Test** |  |
| **17** | **#653** | No | No | n/a | n/a | No |
| **18** | **#654** | No | No | n/a | n/a | No |
| **19** | **#657** | Yes | No | n/a | n/a | No |
| **20** | **#659** | Yes | No | Impaired on right side; aSPV right 10, left 30, warm | Normal, both sides | No |
| **21** | **#660** | No | No | Impaired on right side; aSPV right 2, left 12, warm | Normal, both sides | No |
| **22** | **#663** | No | No | Normal | Normal | No |
| **23** | **#666** | No | No | n/a | n/a | No |
| **24** | **#667** | Yes | No | n/a | n/a | No |
| **25** | **#669** | Yes | No | No reaction on both sides, warm and cold | Pathological, both sides | No |
| **26** | **#670** | No | No | n/a | n/a | No |
| **27** | **#671** | No | No | Normal | Normal | No |
| **28** | **#672** | Yes | No | Normal | Normal | Yes |
| **29** | **#678** | No | No | n/a | n/a | No |
| **30** | **#679** | Yes | No | Left side normal, right side no reaction | Pathological, right side | No |
| **31** | **#680** | No | No | Normal | Normal | No |
| **32** | **#681** | No | No | n/a | n/a | No |

**Table S5: Sanger sequencing results of *GJB2.*** NCBI GeneBank Reference Sequences: NG_008358.1, NM_004004.6, NP_003995.2. The clinical significance is given according to Clin Var (<https://www.ncbi.nlm.nih.gov/clinvar/>). Results of patients 1-16 are taken from our former work (3).

| **Patient** | **Patient ID** | ***GJB2***  ***gDNA*** | | ***GJB2***  ***cDNA*** | | **GJB2**  **protein** | | **SNP ID** | | **Clinical significance** | |
| --- | --- | --- | --- | --- | --- | --- | --- | --- | --- | --- | --- |
|  |  | ***Allele 1*** | ***Allele 2*** | ***Allele 1*** | ***Allele 2*** | ***Allele 1*** | ***Allele 2*** | ***Allele 1*** | ***Allele 2*** | ***Allele 1*** | ***Allele 2*** |
| **1** | **#119** | *WT* | *WT* | *WT* | *WT* | WT | WT |  |  |  |  |
| **2** | **#271** | *WT* | *WT* | *WT* | *WT* | WT | WT |  |  |  |  |
| **3** | **#272** | *WT* | *WT* | *WT* | *WT* | WT | WT |  |  |  |  |
| **4** | **#278** | *WT* | *WT* | *WT* | *WT* | WT | WT |  |  |  |  |
| **5** | **#305** | *WT* | *WT* | *WT* | *WT* | WT | WT |  |  |  |  |
| **6** | **#307** | *g.8429del* | *g.8429del* | *c.35del* | *c.35del* | p.Gly12fs | p.Gly12fs | rs80338939 | rs80338939 | pathogenic | pathogenic |
| **7** | **#358** | *WT* | *WT* | *WT* | *WT* | WT | WT |  |  |  |  |
| **8** | **#359** | *WT* | *WT* | *WT* | *WT* | WT | WT |  |  |  |  |
| **9** | **#395** | *WT* | *WT* | *WT* | *WT* | WT | WT |  |  |  |  |
| **10** | **#421** | *g.8429del* | *g.8429del* | *c.35del* | *c.35del* | p.Gly12fs | p.Gly12fs | rs80338939 | rs80338939 | pathogenic | pathogenic |
| **11** | **#568** | *WT* | *WT* | *WT* | *WT* | WT | WT |  |  |  |  |
| **12** | **#569** | *WT* | *WT* | *WT* | *WT* | WT | WT |  |  |  |  |
| **13** | **#610** | *WT* | *WT* | *WT* | *WT* | WT | WT |  |  |  |  |
| **14** | **#616** | *WT* | *WT* | *WT* | *WT* | WT | WT |  |  |  |  |
| **15** | **#622** | *WT* | *WT* | *WT* | *WT* | WT | WT |  |  |  |  |
| **16** | **#632** | *WT* | *WT* | *WT* | *WT* | WT | WT |  |  |  |  |
| **17** | **#653** | *WT* | *WT* | *WT* | *WT* | WT | WT |  |  |  |  |
| **18** | **#654** | *WT* | *WT* | *WT* | *WT* | WT | WT |  |  |  |  |
| **19** | **#657** | *WT* | *WT* | *WT* | *WT* | WT | WT |  |  |  |  |
| **20** | **#659** | *WT* | *WT* | *WT* | *WT* | WT | WT |  |  |  |  |
| **21** | **#660** | *WT* | *WT* | *WT* | *WT* | WT | WT |  |  |  |  |
| **22** | **#663** | *WT* | *WT* | *WT* | *WT* | WT | WT |  |  |  |  |
| **23** | **#666** | *WT* | *WT* | *WT* | *WT* | WT | WT |  |  |  |  |
| **24** | **#667** | *WT* | *WT* | *WT* | *WT* | WT | WT |  |  |  |  |
| **25** | **#669** | *g.8429del* | *wt* | *c.35del* | *wt* | p.Gly12fs | wt | rs80338939 |  | pathogenic |  |
| **26** | **#670** | *WT* | *WT* | *WT* | *WT* | WT | WT |  |  |  |  |
| **27** | **#671** | *WT* | *WT* | *WT* | *WT* | WT | WT |  |  |  |  |
| **28** | **#672** | *WT* | *WT* | *WT* | *WT* | WT | WT |  |  |  |  |
| **29** | **#678** | *WT* | *WT* | *WT* | *WT* | WT | WT |  |  |  |  |
| **30** | **#679** | *WT* | *WT* | *WT* | *WT* | WT | WT |  |  |  |  |
| **31** | **#680** | *WT* | *WT* | *WT* | *WT* | WT | WT |  |  |  |  |
| **32** | **#681** | *WT* | *WT* | *WT* | *WT* | WT | WT |  |  |  |  |

**Table S6: Sanger sequencing results of *SLC26A4.*** NCBI GeneBank Reference Sequences: NG_008489.1, NM_000441.2, NP_000432.1. The clinical significance is given according to Clin Var (<https://www.ncbi.nlm.nih.gov/clinvar/>). For results of patients 1-16, refer to (3) or Table 2. N.A., not assessed.

| **Patient** | **Patient ID** | ***SLC26A4***  ***gDNA*** | | ***SLC26A4***  ***cDNA*** | | **SLC26A4** | | **SNP ID** | | **Clinical significance** | |
| --- | --- | --- | --- | --- | --- | --- | --- | --- | --- | --- | --- |
|  |  | ***Allele 1*** | ***Allele 2*** | ***Allele 1*** | ***Allele 2*** | ***Allele 1*** | ***Allele 2*** | ***Allele 1*** | ***Allele 2*** | ***Allele 1*** | ***Allele 2*** |
| **17** | **#653** | *g.7543A>T* | *g.7543A>T* | *c.165-119A>T* | *c.165-119A>T* | Intron variant | Intron variant | rs2248464 | rs2248464 | Benign | Benign |
|  |  | *g.7549C>T* | *g.7549C>T* | *c.165-113C>T* | *c.165-113C>T* | Intron variant | Intron variant | rs2248465 | rs2248465 | Benign | Benign |
|  |  | *g.28034G>T* | *WT* | *c.1001+131G>T* | *WT* | Intron variant | WT | rs2395911 |  | Benign |  |
|  |  | *g.47875T>A* | *g.47875T>A* | *c.2035-822T>A* | *c.2035-822T>A* | Intron variant | Intron variant | rs982662 | rs982662 | Not reported | Not reported |
|  |  | *g.48138T>C* | *WT* | *c.2035-559T>C* | *WT* | Intron variant | WT | rs2188562 |  | Not reported |  |
|  |  | *g.54356C>T* | *WT* | *c.2090-64C>T* | *WT* | Intron variant | WT | rs768068176 |  | Not reported |  |
|  |  | *g.54549G>T* | *WT* | *c.2219G>T* | *WT* | p.Gly740Val | WT | rs111033310 |  | Conflicting classifications of pathogenicity:  likely pathogenic/uncertain significance |  |
|  |  | *g.60680G>A* | *WT* | *c.*868G>A* | *WT* | 3´ UTR variant | WT | rs2712218 |  | Benign |  |
| **18** | **#654** | *g.7543A>T* | *g.7543A>T* | *c.165-119A>T* | *c.165-119A>T* | Intron variant | Intron variant | rs2248464 | rs2248464 | Benign | Benign |
|  |  | *g.7549C>T* | *g.7549C>T* | *c.165-113C>T* | *c.165-113C>T* | Intron variant | Intron variant | rs2248465 | rs2248465 | Benign | Benign |
|  |  | *g.28034G>T* | *WT* | *c.1001+131G>T* | *WT* | Intron variant | WT | rs2395911 |  | Benign |  |
|  |  | *g.45549T>C* | *WT* | *c.1790T>C* | *WT* | p.Leu597Ser | WT | rs55638457 |  | Conflicting classifications of pathogenicity:  likely benign/uncertain significance/benign |  |
|  |  | *g.60680G>A* | *WT* | *c.*868G>A* | *WT* | 3´ UTR variant | WT | rs2712218 |  | Benign |  |
| **19** | **#657** | *g.7543A>T* | *g.7543A>T* | *c.165-119A>T* | *c.165-119A>T* | Intron variant | Intron variant | rs2248464 | rs2248464 | Benign |  |
|  |  | *g.7549C>T* | *g.7549C>T* | *c.165-113C>T* | *c.165-113C>T* | Intron variant | Intron variant | rs2248465 | rs2248465 | Benign |  |
|  |  | *g.7887T>C* | *WT* | *g.7887T>C* | *WT* | Intron variant | *WT* | rs1045068050 |  | Not reported |  |
|  |  | *g.19045T>G* | *WT* | *c.601-266T>G* | *WT* | Intron variant | *WT* | rs3817613 |  | Benign |  |
|  |  | *g.28034G>T* | *g.28034G>T* | *c.1001+131G>T* | *c.1001+131G>T* | Intron variant | Intron variant | rs2395911 | rs2395911 | Benign | Benign |
|  |  | *g.40078C>G* | *WT* | N.A. | *WT* | Intron variant | *WT* | N.A. |  | unknown |  |
|  |  | *g.47875T>A* | *g.47875T>A* | *c.2035-822T>A* | *c.2035-822T>A* | Intron variant | Intron variant | rs982662 | rs982662 | Not reported | Not reported |
|  |  | *g.48137C>T* | *WT* | *c.2035-560C>A* | *WT* | Intron variant | WT | rs982664 |  | Not reported |  |
|  |  | *g.48138T>C* | *WT* | *c.2035-559T>C* | *WT* | Intron variant | WT | rs2188562 |  | Not reported |  |
|  |  | *g.48487C>T* | *WT* | *c.2035-210C>T* | *WT* | Intron variant | WT | rs6955309 |  | Benign |  |
| **20** | **#659** | *g.7543A>T* | *g.7543A>T* | *c.165-119A>T* | *c.165-119A>T* | Intron variant | Intron variant | rs2248464 | rs2248464 | Benign | Benign |
|  |  | *g.7549C>T* | *g.7549C>T* | *c.165-113C>T* | *c.165-113C>T* | Intron variant | Intron variant | rs2248465 | rs2248465 | Benign | Benign |
|  |  | *g.16606T>A* | *WT* | *c.407T>A* | *WT* | p.Ile136Asn | *WT* | rs1000929089 |  | Not reported |  |
|  |  | *g.46380C>T* | *WT* | *c.1991C>T* | *WT* | p.Ala664Val | *WT* | rs2129318281 |  | Pathogenic/Likely pathogenic |  |
|  |  | *g.48138T>C* | *WT* | *c.2035-559T>A* | *WT* | Intron variant | *WT* | rs2188562 |  | Not reported |  |
|  |  | *g.60680G>A* | *WT* | *c.*868G>A* | *WT* | 3´ UTR variant | *WT* | rs2712218 |  | Benign |  |
| **21** | **#660** | *g.7543A>T* | *WT* | *c.165-119A>T* | *WT* | Intron variant | *WT* | rs2248464 |  | Benign |  |
|  |  | *g.7549C>T* | *WT* | *c.165-113C>T* | *WT* | Intron variant | *WT* | rs2248465 |  | Benign |  |
|  |  | *g.7799A>G* | *WT* | *c.302A>G* | *WT* | p.Gln101Arg | *WT* | N.A. |  | Unknown |  |
|  |  | *g.19045T>G* | *g.19045T>G* | *c.601-266T>G* | *c.601-266T>G* | Intron variant | Intron variant | rs3817613 | rs3817613 | Benign | Benign |
|  |  | *g.28034G>T* | *g.28034G>T* | *c.1001+131G>T* | *c.1001+131G>T* | Intron variant | Intron variant | rs2395911 | rs2395911 | Benign | Benign |
|  |  | *g.34566G>A* | *WT* | *c.1226G>A* | *WT* | p.Arg409His | *WT* | rs111033305 |  | Pathogenic/Likely pathogenic |  |
|  |  | *g.39979C>A* | *WT* | *c.1438-320C>A* | *WT* | Intron variant | *WT* | rs2188561 |  | Benign |  |
|  |  | *g.47875T>A* | *g.47875T>A* | *c.2035-822T>A* | *c.2035-822T>A* | Intron variant | Intron variant | rs982662 | rs982662 | Not reported | Not reported |
|  |  | *g.48138T>C* | *WT* | *c.2035-559T>A* | *WT* | Intron variant | *WT* | rs2188562 |  | Not reported |  |
|  |  | *g.60680G>A* | *WT* | *c.*868G>A* | *WT* | 3´ UTR variant | *WT* | rs2712218 |  | Benign |  |
| **22** | **#663** | *g.7543A>T* | *WT* | *c.165-119A>T* | *WT* | Intron variant | *WT* | rs2248464 |  | Benign |  |
|  |  | *g.7549C>T* | *WT* | *c.165-113C>T* | *WT* | Intron variant | *WT* | rs2248465 |  | Benign |  |
|  |  | *g.19045T>G* | *WT* | *c.601-266T>G* | *WT* | Intron variant | *WT* | rs3817613 |  | Benign |  |
|  |  | *g.48487C>T* | *WT* | *c.2035-210C>T* | *WT* | Intron variant | *WT* | rs6955309 |  | Benign |  |
|  |  | *g.54356C>T* | *WT* | *c.2090-64C>T* | *WT* | Intron variant | *WT* | rs768068176 |  | Not reported |  |
|  |  | *g.60680G>A* | *WT* | *c.*868G>A* | *WT* | 3´ UTR variant | *WT* | rs2712218 |  | Benign |  |
| **23** | **#666** | *g.7543A>T* | *WT* | *c.165-119A>T* | *WT* | Intron variant | *WT* | rs2248464 |  | Benign |  |
|  |  | *g.7549C>T* | *WT* | *c.165-113C>T* | *WT* | Intron variant | *WT* | rs2248465 |  | Benign |  |
|  |  | *g.19045T>G* | *WT* | *c.601-266T>G* | *WT* | Intron variant | *WT* | rs3817613 |  | Benign |  |
|  |  | *g.48137C>T* | *g.48137C>T* | *c.2035-560C>A* | *c.2035-560C>A* | Intron variant | Intron variant | rs982664 | rs982664 | Not reported | Not reported |
|  |  | *g.48138T>C* | *WT* | *c.2035-559T>A* | *WT* | Intron variant | *WT* | rs2188562 |  | Not reported |  |
|  |  | *g.48487C>T* | *WT* | *c.2035-210C>T* | *WT* | Intron variant | *WT* | rs6955309 |  | Benign |  |
| **24** | **#667** | *g.5122T>C* | *WT* | *c.-103T>C* | *WT* | 5´ UTR variant | *WT* | rs60284988 |  | Uncertain significance |  |
|  |  | *g.5452T>C* | *WT* | *c.-4+231T>C* | *WT* | Intron variant | *WT* | rs546225214 |  | Not reported |  |
|  |  | *g.7543A>T* | *g.7543A>T* | *c.165-119A>T* | *c.165-119A>T* | Intron variant | Intron variant | rs2248464 | rs2248464 | Benign | Benign |
|  |  | *g.7549C>T* | *g.7549C>T* | *c.165-113C>T* | *c.165-113C>T* | Intron variant | Intron variant | rs2248465 | rs2248465 | Benign | Benign |
|  |  | *g.18828C>T* | *WT* | *c.600+114C>T* | *WT* | Intron variant | *WT* | rs1327200615 |  | Not reported |  |
|  |  | *g.19045T>G* | *WT* | *c.601-266T>G* | *WT* | Intron variant | *WT* | rs3817613 |  | Benign |  |
|  |  | *g.34481C>T* | *WT* | *c.1150-9C>T* | *WT* | Intron variant | *WT* | rs780920429 |  | Likely benign |  |
|  |  | *g.48137C>T* | *WT* | *c.2035-560C>A* | *WT* | Intron variant | WT | rs982664 |  | Not reported |  |
|  |  | *g.48138T>C* | *g.48138T>C* | *c.2035-559T>A* | *c.2035-559T>A* | Intron variant | Intron variant | rs2188562 | rs2188562 | Not reported | Not reported |
|  |  | *g.48487C>T* | *WT* | *c.2035-210C>T* | *WT* | Intron variant | *WT* | rs6955309 |  | Benign |  |
|  |  | *g.60680G>A* | *WT* | *c.*868G>A* | *WT* | 3´ UTR variant | *WT* | rs2712218 |  | Benign |  |
|  |  | *g.60935A>G* | *WT* | *c.*1123A>G* | *WT* | 3´ UTR variant | *WT* | rs141341508 |  | Uncertain significance |  |
| **25** | **#669** | *g.7543A>T* | *g.7543A>T* | *c.165-119A>T* | *c.165-119A>T* | Intron variant | Intron variant | rs2248464 | rs2248464 | Benign | Benign |
|  |  | *g.7549C>T* | *g.7549C>T* | *c.165-113C>T* | *c.165-113C>T* | Intron variant | Intron variant | rs2248465 | rs2248465 | Benign | Benign |
|  |  | *g.19045T>G* | *WT* | *c.601-266T>G* | *WT* | Intron variant | *WT* | rs3817613 |  | Benign |  |
|  |  | *g.28034G>T* | *g.28034G>T* | *c.1001+131G>T* | *c.1001+131G>T* | Intron variant | Intron variant | rs2395911 | rs2395911 | Benign | Benign |
|  |  | *g.47875T>A* | *g.47875T>A* | *c.2035-822T>A* | *c.2035-822T>A* | Intron variant | Intron variant | rs982662 | rs982662 | Not reported | Not reported |
|  |  | *g.48137C>T* | *WT* | *c.2035-560C>A* | *WT* | Intron variant | WT | rs982664 |  | Not reported |  |
|  |  | *g.48138T>C* | *g.48138T>C* | *c.2035-559T>A* | *c.2035-559T>A* | Intron variant | Intron variant | rs2188562 | rs2188562 | Not reported | Not reported |
|  |  | *g.48487C>T* | *WT* | *c.2035-210C>T* | *WT* | Intron variant | *WT* | rs6955309 |  | Benign |  |
|  |  | *g.60680G>A* | *WT* | *c.*868G>A* | *WT* | 3´ UTR variant | *WT* | rs2712218 |  | Benign |  |
| **26** | **#670** | *g.7543A>T* | *WT* | *c.165-119A>T* | *WT* | Intron variant | *WT* | rs2248464 |  | Benign |  |
|  |  | *g.7549C>T* | *WT* | *c.165-113C>T* | *WT* | Intron variant | *WT* | rs2248465 |  | Benign |  |
|  |  | *g.18668G>C* | *WT* | *c.554G>C* | *WT* | p.Arg185Thr | *WT* | rs542620119 |  | Likely pathogenic |  |
|  |  | *g.19454delT* | *WT* | *c.744delT* | *WT* | p.Asn248Lysfs*41 | *WT* | N.A. |  | Unknown |  |
|  |  | *g.48487C>T* | *WT* | *c.2035-210C>T* | *WT* | Intron variant | *WT* | rs6955309 |  | Benign |  |
|  |  | *g.60132A>G* | *WT* | *c.*320=* | *WT* | 3´ UTR variant | *WT* | rs1242683086 |  | Not reported |  |
| **27** | **#671** | *g.7543A>T* | *g.7543A>T* | *c.165-119A>T* | *c.165-119A>T* | Intron variant | Intron variant | rs2248464 | rs2248464 | Benign | Benign |
|  |  | *g.7549C>T* | *g.7549C>T* | *c.165-113C>T* | *c.165-113C>T* | Intron variant | Intron variant | rs2248465 | rs2248465 | Benign | Benign |
|  |  | *g.27904G>A* | *WT* | *c.1001+1G>A* | *WT* | Splice donor variant | *WT* | rs80338849 |  | Pathogenic |  |
|  |  | *g.34355C>T* | *WT* | *c.1150-135C>T* | *WT* | Intron variant | *WT* | rs2072063 |  | Benign |  |
|  |  | *g.45449T>A* | *WT* | *c.1708-18T>A* | *WT* | Intron variant | *WT* | rs55701254 |  | Benign |  |
|  |  | *g.48138T>C* | *g.48138T>C* | *c.2035-559T>A* | *c.2035-559T>A* | Intron variant | Intron variant | rs2188562 | rs2188562 | Not reported | Not reported |
|  |  | *g.60680G>A* | *WT* | *c.*868G>A* | *WT* | 3´ UTR variant | *WT* | rs2712218 |  | Benign |  |
|  |  | *g.60871T>C* | *WT* | *c.*1059T>C* | *WT* | 3´ UTR variant | *WT* | rs183926745 |  | Uncertain significance |  |
| **28** | **#672** | *g.7543A>T* | *g.7543A>T* | *c.165-119A>T* | *c.165-119A>T* | Intron variant | Intron variant | rs2248464 | rs2248464 | Benign | Benign |
|  |  | *g.7549C>T* | *g.7549C>T* | *c.165-113C>T* | *c.165-113C>T* | Intron variant | Intron variant | rs2248465 | rs2248465 | Benign | Benign |
|  |  | *g.19045T>G* | *g.19045T>G* | *c.601-266T>G* | *c.601-266T>G* | Intron variant | Intron variant | rs3817613 | rs3817613 | Benign | Benign |
|  |  | *g.48137C>T* | *g.48137C>T* | *c.2035-560C>A* | *c.2035-560C>A* | Intron variant | Intron variant | rs982664 | rs982664 | Not reported | Not reported |
|  |  | *g.48138T>C* | *g.48138T>C* | *c.2035-559T>A* | *c.2035-559T>A* | Intron variant | Intron variant | rs2188562 | rs2188562 | Not reported | Not reported |
|  |  | *g.48487C>T* | *g.48487C>T* | *c.2035-210C>T* | *c.2035-210C>T* | Intron variant | Intron variant | rs6955309 | rs6955309 | Benign | Benign |
| **29** | **#678** | *g.5744C>T* | *WT* | *c.-3-261C>T* | *WT* | Intron variant | *WT* | rs55753070 |  | Likely benign |  |
|  |  | *g.19045T>G* | *g.19045T>G* | *c.601-266T>G* | *c.601-266T>G* | Intron variant | Intron variant | rs3817613 | rs3817613 | Benign | Benign |
|  |  | *g.48487C>T* | *g.48487C>T* | *c.2035-210C>T* | *c.2035-210C>T* | Intron variant | Intron variant | rs6955309 | rs6955309 | Benign | Benign |
| **30** | **#679** | *g.5271C>T* | *WT* | *c.-4+50C>A* | *WT* | Intron variant | *WT* | rs912566709 |  | Not reported |  |
|  |  | *g.5896dupG* | *g.5896dupG* | *c.-3-109dup* | *c.-3-109dup* | Intron variant | Intron variant | rs200483647 | rs200483647 | Likely benign | Likely benign |
|  |  | *g.7543A>T* | *g.7543A>T* | *c.165-119A>T* | *c.165-119A>T* | Intron variant | Intron variant | rs2248464 | rs2248464 | Benign | Benign |
|  |  | *g.7549C>T* | *g.7549C>T* | *c.165-113C>T* | *c.165-113C>T* | Intron variant | Intron variant | rs2248465 | rs2248465 | Benign | Benign |
|  |  | *g.17868G>A* | *g.17868G>A* | *c.416-662G>A* | *c.416-662G>A* | Intron variant | Intron variant | rs11769313 | rs11769313 | Not reported | Not reported |
|  |  | *g.19045T>G* | *g.19045T>G* | *c.601-266T>G* | *c.601-266T>G* | Intron variant | Intron variant | rs3817613 | rs3817613 | Benign | Benign |
| **31** | **#680** | *g.7543A>T* | *g.7543A>T* | *c.165-119A>T* | *c.165-119A>T* | Intron variant | Intron variant | rs2248464 | rs2248464 | Benign | Benign |
|  |  | *g.7549C>T* | *g.7549C>T* | *c.165-113C>T* | *c.165-113C>T* | Intron variant | Intron variant | rs2248465 | rs2248465 | Benign | Benign |
|  |  | *g.19045T>G* | *WT* | *c.601-266T>G* | *WT* | Intron variant | *WT* | rs3817613 |  | Benign |  |
|  |  | *g.48137C>T* | *WT* | *c.2035-560C>A* | *WT* | Intron variant | *WT* | rs982664 |  | Not reported |  |
|  |  | *g.48138T>C* | *g.48138T>C* | *c.2035-559T>A* | *c.2035-559T>A* | Intron variant | Intron variant | rs2188562 | rs2188562 | Not reported | Not reported |
|  |  | *g.48487C>T* | *WT* | *c.2035-210C>T* | *WT* | Intron variant | *WT* | rs6955309 |  | Benign |  |
|  |  | *g.60162C>T* | *WT* | *c.*350C>T* | *WT* | 3´ UTR variant | *WT* | rs17154362 |  | Conflicting classifications of pathogenicity: Uncertain significance; Likely benign |  |
|  |  | *g.60680G>A* | *WT* | *c.*868G>A* | *WT* | 3´ UTR variant | *WT* | rs2712218 |  | Benign |  |
| **32** | **#681** | *g.7543A>T* | *g.7543A>T* | *c.165-119A>T* | *c.165-119A>T* | Intron variant | Intron variant | rs2248464 | rs2248464 | Benign | Benign |
|  |  | *g.7549C>T* | *g.7549C>T* | *c.165-113C>T* | *c.165-113C>T* | Intron variant | Intron variant | rs2248465 | rs2248465 | Benign | Benign |
|  |  | *g.7730A>G* | *g.7730A>G* | *c.233A>G* | *c.233A>G* | p.Tyr78Cys | p.Tyr78Cys | rs2129309178 | rs2129309178 | Not reported | Not reported |
|  |  | *g.19045T>G* | *g.19045T>G* | *c.601-266T>G* | *c.601-266T>G* | Intron variant | Intron variant | rs3817613 | rs3817613 | Benign | Benign |
|  |  | *g.48137C>T* | *g.48137C>T* | *c.2035-560C>A* | *c.2035-560C>A* | Intron variant | Intron variant | rs982664 | rs982664 | Not reported | Not reported |
|  |  | *g.48138T>C* | *g.48138T>C* | *c.2035-559T>A* | *c.2035-559T>A* | Intron variant | Intron variant | rs2188562 | rs2188562 | Not reported | Not reported |
|  |  | *g.48487C>T* | *g.48487C>T* | *c.2035-210C>T* | *c.2035-210C>T* | Intron variant | Intron variant | rs6955309 | rs6955309 | Benign | Benign |

**Table S7: Sanger sequencing results of *FOXI1*.** NCBI GeneBank Reference Sequence: NG_012068.1, NM_012188.5, NP_036320.2. The clinical significance is given according to Clin Var (https://www.ncbi.nlm.nih.gov/clinvar/).

|  | **Patient ID** | ***FOXI1***  ***gDNA*** | | ***FOXI1***  ***cDNA*** | | **FOXI1** | | **SNP ID** | | **Clinical significance** | |
| --- | --- | --- | --- | --- | --- | --- | --- | --- | --- | --- | --- |
|  |  | ***Allele 1*** | ***Allele 2*** | ***Allele 1*** | ***Allele 2*** | **Allele 1** | **Allele 2** | ***Allele 1*** | ***Allele 2*** | ***Allele 1*** | ***Allele 2*** |
| **1** | **#119** | *g.5324G>A* | *WT* | *c.279G>A* | *WT* | p.Arg93 | WT | rs2277944 |  | Benign |  |
|  |  | *g.5690T>C* | *WT* | *c.574+71T>C* | *WT* | intron variant | WT | rs2277945 |  | Benign |  |
|  |  | *g.7606T>C* | *g.7606T>C* | *c.1044T>C* | *c.1044T>C* | p.Tyr348 | p.Tyr348 | rs10063424 | rs10063424 | Benign | Benign |
| **2** | **#271** | *g.5324G>A* | *WT* | *c.279G>A* | *WT* | p.Arg93 | WT | rs2277944 |  | Benign |  |
|  |  | *g.7606T>C* | *g.7606T>C* | *c.1044T>C* | *c.1044T>C* | p.Tyr348 | p.Tyr348 | rs10063424 | rs10063424 | Benign | Benign |
| **3** | **#272** | *g.5324G>A* | *g.5324G>A* | *c.279G>A* | *c.279G>A* | p.Arg93 | p.Arg93 | rs2277944 | rs2277944 | Benign | Benign |
|  |  | *g.7606T>C* | *g.7606T>C* | *c.1044T>C* | *c.1044T>C* | p.Tyr348 | p.Tyr348 | rs10063424 | rs10063424 | Benign | Benign |
| **4** | **#278** | *g.5324G>A* | *WT* | *c.279G>A* | *WT* | p.Arg93 | WT | rs2277944 |  | Benign |  |
|  |  | *g.5690T>C* | *WT* | *c.574+71T>C* | *WT* | intron variant | WT | rs2277945 |  | Benign |  |
|  |  | *g.7606T>C* | *g.7606T>C* | *c.1044T>C* | *c.1044T>C* | p.Tyr348 | p.Tyr348 | rs10063424 | rs10063424 | Benign | Benign |
| **5** | **#305** | *g.5324G>A* | *WT* | *c.279G>A* | *WT* | p.Arg93 | WT | rs2277944 |  | Benign |  |
|  |  | *g.7606T>C* | *g.7606T>C* | *c.1044T>C* | *c.1044T>C* | p.Tyr348 | p.Tyr348 | rs10063424 | rs10063424 | Benign | Benign |
| **6** | **#307** | *g.5690T>C* | *g.5690T>C* | *c.574+71T>C* | *c.574+71T>C* | intron variant | intron variant | rs2277945 | rs2277945 | Benign | Benign |
|  |  | *g.7534G>C* | *WT* | *c.972G>C* | *WT* | p.Pro324 | WT | rs56128152 |  | Conflicting classifications of pathogenicity:  uncertain significance/benign |  |
|  |  | *g.7606T>C* | *g.7606T>C* | *c.1044T>C* | *c.1044T>C* | p.Tyr348 | p.Tyr348 | rs10063424 | rs10063424 | Benign | Benign |
| **7** | **#358** | *g.5324G>A* | *g.5324G>A* | *c.279G>A* | *c.279G>A* | p.Arg93 | p.Arg93 | rs2277944 | rs2277944 | Benign | Benign |
|  |  | *g.7606T>C* | *g.7606T>C* | *c.1044T>C* | *c.1044T>C* | p.Tyr348 | p.Tyr348 | rs10063424 | rs10063424 | Benign | Benign |
| **8** | **#359** | *g.5324G>A* | *WT* | *c.279G>A* | *WT* | p.Arg93 | WT | rs2277944 |  | Benign |  |
|  |  | *g.7606T>C* | *g.7606T>C* | *c.1044T>C* | *c.1044T>C* | p.Tyr348 | p.Tyr348 | rs10063424 | rs10063424 | Benign | Benign |
| **9** | **#395** | *g.5324G>A* | *WT* | *c.279G>A* | *WT* | p.Arg93 | WT | rs2277944 |  | Benign |  |
|  |  | *g.5690T>C* | *WT* | *c.574+71T>C* | *WT* | intron variant | WT | rs2277945 |  | Benign |  |
|  |  | *g.7606T>C* | *g.7606T>C* | *c.1044T>C* | *c.1044T>C* | p.Tyr348 | p.Tyr348 | rs10063424 | rs10063424 | Benign | Benign |
| **10** | **#421** | *g.5690T>C* | *WT* | *c.574+71T>C* | *WT* | intron variant | WT | rs2277945 |  | Benign |  |
|  |  | *g.5719C>A* | *WT* | *c.574+100C>A* | *WT* | intron variant | WT | rs56355825 |  | Not reported |  |
|  |  | *g.7606T>C* | *g.7606T>C* | *c.1044T>C* | *c.1044T>C* | p.Tyr348 | p.Tyr348 | rs10063424 | rs10063424 | Benign | Benign |
| **11** | **#568** | *g.5690T>C* | *WT* | *c.574+71T>C* | *WT* | intron variant | WT | rs2277945 |  | Benign |  |
|  |  | *g.7606T>C* | *g.7606T>C* | *c.1044T>C* | *c.1044T>C* | p.Tyr348 | p.Tyr348 | rs10063424 | rs10063424 | Benign | Benign |
| **12** | **#569** | *g.5690T>C* | *WT* | *c.574+71T>C* | *WT* | intron variant | WT | rs2277945 |  | Benign |  |
|  |  | *g.7606T>C* | *g.7606T>C* | *c.1044T>C* | *c.1044T>C* | p.Tyr348 | p.Tyr348 | rs10063424 | rs10063424 | Benign | Benign |
| **13** | **#610** | *g.5324G>A* | *WT* | *c.279G>A* | *WT* | p.Arg93 | WT | rs2277944 |  | Benign |  |
|  |  | *g.7576G>A* | *WT* | *c.1014G>A* | *WT* | p.Ala338 | WT | rs55685928 |  | Benign/likely benign |  |
|  |  | *g.7606T>C* | *WT* | *c.1044T>C* | *WT* | p.Tyr348 | WT | rs10063424 |  | Benign |  |
| **14** | **#616** | *g.5690T>C* | *WT* | *c.574+71T>C* | *WT* | intron variant | WT | rs2277945 |  | Benign |  |
|  |  | *g.7606T>C* | *g.7606T>C* | *c.1044T>C* | *c.1044T>C* | p.Tyr348 | p.Tyr348 | rs10063424 | rs10063424 | Benign | Benign |
| **15** | **#622** | *g.5690T>C* | *WT* | *c.574+71T>C* | *WT* | intron variant | WT | rs2277945 |  | Benign |  |
|  |  | *g.7606T>C* | *g.7606T>C* | *c.1044T>C* | *c.1044T>C* | p.Tyr348 | p.Tyr348 | rs10063424 | rs10063424 | Benign | Benign |
| **16** | **#632** | *g.5671C>T* | *WT* | *c.574+52C>T* | *WT* | intron variant | WT | rs56128217 |  | Not reported |  |
|  |  | *g.5690T>C* | *WT* | *c.574+71T>C* | *WT* | intron variant | WT | rs2277945 |  | Benign |  |
|  |  | *g.5719C>A* | *WT* | *c.574+100C>A* | *WT* | intron variant | WT | rs56355825 |  | Not reported |  |
|  |  | *g.7606T>C* | *g.7606T>C* | *c.1044T>C* | *c.1044T>C* | p.Tyr348 | p.Tyr348 | rs10063424 | rs10063424 | Benign | Benign |
| **17** | **#653** | *g.5690T>C* | *g.5690T>C* | *c.574+71T>C* | *c.574+71T>C* | intron variant | intron variant | rs2277945 | rs2277945 | Benign | Benign |
|  |  | *g.7606T>C* | *g.7606T>C* | *c.1044T>C* | *c.1044T>C* | p.Tyr348 | p.Tyr348 | rs10063424 | rs10063424 | Benign | Benign |
| **18** | **#654** | *g.5324G>A* | *WT* | *c.279G>A* | *WT* | p.Arg93 | WT | rs2277944 |  | Benign |  |
|  |  | *g.5690T>C* | *g.5690T>C* | *c.574+71T>C* | *c.574+71T>C* | intron variant | intron variant | rs2277945 | rs2277945 | Benign | Benign |
|  |  | *g.7606T>C* | *g.7606T>C* | *c.1044T>C* | *c.1044T>C* | p.Tyr348 | p.Tyr348 | rs10063424 | rs10063424 | Benign | Benign |
| **19** | **#657** | *g.5690T>C* | *g.5690T>C* | *c.574+71T>C* | *c.574+71T>C* | intron variant | intron variant | rs2277945 | rs2277945 | Benign | Benign |
|  |  | *g.7606T>C* | *g.7606T>C* | *c.1044T>C* | *c.1044T>C* | p.Tyr348 | p.Tyr348 | rs10063424 | rs10063424 | Benign | Benign |
| **20** | **#659** | *g.7606T>C* | *g.7606T>C* | *c.1044T>C* | *c.1044T>C* | p.Tyr348 | p.Tyr348 | rs10063424 | rs10063424 | Benign | Benign |
| **21** | **#660** | *g.5719C>A* | *WT* | *c.574+100C>A* | *WT* | intron variant | WT | rs56355825 |  | Not reported |  |
|  |  | *g.7606T>C* | *g.7606T>C* | *c.1044T>C* | *c.1044T>C* | p.Tyr348 | p.Tyr348 | rs10063424 | rs10063424 | Benign | Benign |
| **22** | **#663** | *g.5324G>A* | *WT* | *c.279G>A* | *WT* | p.Arg93 | WT | rs2277944 |  | Benign |  |
|  |  | *g.7606T>C* | *g.7606T>C* | *c.1044T>C* | *c.1044T>C* | p.Tyr348 | p.Tyr348 | rs10063424 | rs10063424 | Benign | Benign |
| **23** | **#666** | *g.5690T>C* | *g.5690T>C* | *c.574+71T>C* | *c.574+71T>C* | intron variant | intron variant | rs2277945 | rs2277945 | Benign | Benign |
|  |  | *g.7606T>C* | *g.7606T>C* | *c.1044T>C* | *c.1044T>C* | p.Tyr348 | p.Tyr348 | rs10063424 | rs10063424 | Benign | Benign |
| **24** | **#667** | *g.7606T>C* | *g.7606T>C* | *c.1044T>C* | *c.1044T>C* | p.Tyr348 | p.Tyr348 | rs10063424 | rs10063424 | Benign | Benign |
| **25** | **#669** | *g.5324G>A* | *WT* | *c.279G>A* | *WT* | p.Arg93 | WT | rs2277944 |  | Benign |  |
|  |  | *g.7606T>C* | *WT* | *c.1044T>C* | *WT* | p.Tyr348 | WT | rs10063424 |  | Benign |  |
| **26** | **#670** | *g.7606T>C* | *g.7606T>C* | *c.1044T>C* | *c.1044T>C* | p.Tyr348 | p.Tyr348 | rs10063424 | rs10063424 | Benign | Benign |
| **27** | **#671** | *g.5690T>C* | *g.5690T>C* | *c.574+71T>C* | *c.574+71T>C* | intron variant | intron variant | rs2277945 | rs2277945 | Benign | Benign |
|  |  | *g.7606T>C* | *g.7606T>C* | *c.1044T>C* | *c.1044T>C* | p.Tyr348 | p.Tyr348 | rs10063424 | rs10063424 | Benign | Benign |
| **28** | **#672** | *g.5690T>C* | *WT* | *c.574+71T>C* | *WT* | intron variant | WT | rs2277945 |  | Benign |  |
|  |  | *g.7606T>C* | *g.7606T>C* | *c.1044T>C* | *c.1044T>C* | p.Tyr348 | p.Tyr348 | rs10063424 | rs10063424 | Benign | Benign |
| **29** | **#678** | *g.5324G>A* | *WT* | *c.279G>A* | *WT* | p.Arg93 | WT | rs2277944 |  | Benign |  |
|  |  | *g.7288C>T* | *WT* | *c.726C>T* | *WT* | p.Ser242 | WT | rs35678180 |  | Conflicting classifications of pathogenicity:  uncertain significance/benign/likely benign |  |
|  |  | *g.7606T>C* | *WT* | *c.1044T>C* | *WT* | p.Tyr348 | WT | rs10063424 |  | Benign |  |
| **30** | **#679** | *g.5324G>A* | *WT* | *c.279G>A* | *WT* | p.Arg93 | WT | rs2277944 |  | Benign |  |
|  |  | *g.5690T>C* | *WT* | *c.574+71T>C* | *WT* | intron variant | WT | rs2277945 |  | Benign |  |
|  |  | *g.7606T>C* | *g.7606T>C* | *c.1044T>C* | *c.1044T>C* | p.Tyr348 | p.Tyr348 | rs10063424 | rs10063424 | Benign | Benign |
| **31** | **#680** | *g.5719C>A* | *WT* | *c.574+100C>A* | *WT* | intron variant | WT | rs56355825 |  | Not reported |  |
|  |  | *g.7606T>C* | *WT* | *c.1044T>C* | *WT* | p.Tyr348 | WT | rs10063424 |  | Benign |  |
| **32** | **#681** | *g.5690T>C* | *WT* | *c.574+71T>C* | *WT* | intron variant | WT | rs2277945 |  | Benign |  |
|  |  | *g.7606T>C* | *g.7606T>C* | *c.1044T>C* | *c.1044T>C* | p.Tyr348 | p.Tyr348 | rs10063424 | rs10063424 | Benign | Benign |

**Table S8: Sanger sequencing results of *KCNJ10.*** NCBI GeneBank Reference Sequences: NG_016411.1, NM_002241.5***,*** NP_002232.2. The clinical significance is given according to Clin Var (<https://www.ncbi.nlm.nih.gov/clinvar/>).

| **Patient** | **Patient ID** | ***KCNJ10***  ***gDNA*** | | ***KCNJ10***  ***cDNA*** | | **KCNJ10**  **protein** | | **SNP ID** | | **Clinical significance** | |
| --- | --- | --- | --- | --- | --- | --- | --- | --- | --- | --- | --- |
|  |  | ***Allele 1*** | ***Allele 2*** | ***Allele 1*** | ***Allele 2*** | ***Allele 1*** | ***Allele 2*** | ***Allele 1*** | ***Allele 2*** | ***Allele 1*** | ***Allele 2*** |
| **1** | **#119** | *WT* | *WT* | *WT* | *WT* | WT | WT |  |  |  |  |
| **2** | **#271** | *WT* | *WT* | *WT* | *WT* | WT | WT |  |  |  |  |
| **3** | **#272** | *WT* | *WT* | *WT* | *WT* | WT | WT |  |  |  |  |
| **4** | **#278** | *WT* | *WT* | *WT* | *WT* | WT | WT |  |  |  |  |
| **5** | **#305** | *g.33450C>T* | *g.33450C>T* | *c.811C>T* | *c.811C>T* | p.Arg271Cys | p.Arg271Cys | rs1130183 | rs1130183 | Benign/likely benign | Benign/likely benign |
| **6** | **#307** | *WT* | *WT* | *WT* | *WT* | WT | WT |  |  |  |  |
| **7** | **#358** | *WT* | *WT* | *WT* | *WT* | WT | WT |  |  |  |  |
| **8** | **#359** | *WT* | *WT* | *WT* | *WT* | WT | WT |  |  |  |  |
| **9** | **#395** | *WT* | *WT* | *WT* | *WT* | WT | WT |  |  |  |  |
| **10** | **#421** | *WT* | *WT* | *WT* | *WT* | WT | WT |  |  |  |  |
| **11** | **#568** | *WT* | *WT* | *WT* | *WT* | WT | WT |  |  |  |  |
| **12** | **#569** | *g.33450C>T* | *WT* | *c.811C>T* | *WT* | p.Arg271Cys | WT | rs1130183 |  | Benign/likely benign |  |
| **13** | **#610** | *WT* | *WT* | *WT* | *WT* | WT | WT |  |  |  |  |
| **14** | **#616** | *g.32692G>A* | *WT* | *c.53G>A* | *WT* | p.Arg18Gln | WT | rs115466046 |  | Conflicting classifications of pathogenicity: Uncertain significance/benign/likely benign |  |
| **15** | **#622** | *WT* | *WT* | *WT* | *WT* | WT | WT |  |  |  |  |
| **16** | **#632** | *WT* | *WT* | *WT* | *WT* | WT | WT |  |  |  |  |
| **17** | **#653** | *g.33450C>T* | *WT* | *c.811C>T* | *WT* | p.Arg271Cys | WT | rs1130183 |  | Benign/likely benign |  |
| **18** | **#654** | *WT* | *WT* | *WT* | *WT* | WT | WT | *WT* |  |  |  |
| **19** | **#657** | *g.33450C>T* | *WT* | *c.811C>T* | *WT* | p.Arg271Cys | WT | rs1130183 |  | Benign/likely benign |  |
| **20** | **#659** | *WT* | *WT* | *WT* | *WT* | WT | WT | *WT* |  |  |  |
| **21** | **#660** | *g.33169A>G* | *WT* | *c.530A>G* | *WT* | p.Glu177Gly | WT | rs145947380 |  | Conflicting classifications of pathogenicity: Uncertain significance/benign/likely benign |  |
| **22** | **#663** | *WT* | *WT* | *WT* | *WT* | WT | WT |  |  |  |  |
| **23** | **#666** | *WT* | *WT* | *WT* | *WT* | WT | WT |  |  |  |  |
| **24** | **#667** | *WT* | *WT* | *WT* | *WT* | WT | WT |  |  |  |  |
| **25** | **#669** | *WT* | *WT* | *WT* | *WT* | WT | WT |  |  |  |  |
| **26** | **#670** | *WT* | *WT* | *WT* | *WT* | WT | WT |  |  |  |  |
| **27** | **#671** | *WT* | *WT* | *WT* | *WT* | WT | WT |  |  |  |  |
| **28** | **#672** | *WT* | *WT* | *WT* | *WT* | WT | WT |  |  |  |  |
| **29** | **#678** | *WT* | *WT* | *WT* | *WT* | WT | WT |  |  |  |  |
| **30** | **#679** | *WT* | *WT* | *WT* | *WT* | WT | WT |  |  |  |  |
| **31** | **#680** | *WT* | *WT* | *WT* | *WT* | WT | WT |  |  |  |  |
| **32** | **#681** | *WT* | *WT* | *WT* | *WT* | WT | WT |  |  |  |  |

**Table S9: Sanger sequencing results of *GJB3*.** NCBI GeneBank Reference Sequences: NG_008309.1, NM_024009.3, NP_076872.1. The clinical significance is given according to Clin Var (<https://www.ncbi.nlm.nih.gov/clinvar/>).

| **Patient** | **Patient ID** | **GJB3**  **gDNA** | | **GJB3**  **cDNA** | | **GJB3**  **protein** | | **SNP ID** | | **Clinical significance** | |
| --- | --- | --- | --- | --- | --- | --- | --- | --- | --- | --- | --- |
|  |  | ***Allele 1*** | ***Allele 2*** | ***Allele 1*** | ***Allele 2*** | ***Allele 1*** | ***Allele 2*** | **Allele 1** | **Allele 2** | **Allele 1** | **Allele 2** |
| **1** | **#119** | *g.8668C>T* | *WT* | *c.94C>T* | *WT* | p.Arg32Trp | WT | rs1805063 |  | Benign |  |
|  |  | *g.9430C>A* | *WT* | **43C>A* | *WT* | 3’ UTR variant | *WT* | rs41266429 |  | Benign |  |
|  |  | *g.9440G>A* | *g.9440G>A* | *c.*53G>A* | *c.*53G>A* | 3’ UTR variant | 3’ UTR variant | rs476220 | rs476220 | Benign | Benign |
| **2** | **#271** | *WT* | *WT* | *WT* | *WT* | WT | WT |  |  |  |  |
| **3** | **#272** | *g.9440G>A* | *WT* | *c.*53G>A* | *WT* | 3’ UTR variant | *WT* | rs476220 |  | Benign |  |
| **4** | **#278** | *WT* | *WT* | *WT* | *WT* | WT | WT |  |  |  |  |
| **5** | **#305** | *g.9440G>A* | *WT* | *c.*53G>A* | *WT* | 3’ UTR variant | *WT* | rs476220 |  | Benign |  |
| **6** | **#307** | *g.9372C>T* | *WT* | *c.798C>T* | *WT* | p.Asn266Asn | WT | rs35983826 |  | Benign |  |
| **7** | **#358** | *g.8668C>T* | *WT* | *c.94C>T* | *WT* | p.Arg32Trp | WT | rs1805063 |  | Benign |  |
|  |  | *g.9440G>A* | *WT* | *c.*53G>A* | *WT* | 3’ UTR variant | *WT* | rs476220 |  | Benign |  |
| **8** | **#359** | *g.9440G>A* | *g.9440G>A* | *c.*53G>A* | *c.*53G>A* | 3’ UTR variant | 3’ UTR variant | rs476220 | rs476220 | Benign | Benign |
| **9** | **#395** | *g.9430C>A* | *WT* | **43C>A* | *WT* | 3’ UTR variant | *WT* | rs41266429 |  | Benign |  |
|  |  | *g.9440G>A* | *g.9440G>A* | *c.*53G>A* | *c.*53G>A* | 3’ UTR variant | 3’ UTR variant | rs476220 | rs476220 | Benign | Benign |
| **10** | **#421** | *g.9372C>T* | *WT* | *c.798C>T* | *WT* | p.Asn266Asn | WT | rs35983826 |  | Benign |  |
|  |  | *g.9440G>A* | *g.9440G>A* | *c.*53G>A* | *c.*53G>A* | 3’ UTR variant | 3’ UTR variant | rs476220 | rs476220 | Benign | Benign |
| **11** | **#568** | *g.9440G>A* | *g.9440G>A* | *c.*53G>A* | *c.*53G>A* | 3’ UTR variant | 3’ UTR variant | rs476220 | rs476220 | Benign | Benign |
| **12** | **#569** | *g.9372C>T* | *WT* | *c.798C>T* | *WT* | p.Asn266Asn | WT | rs35983826 |  | Benign |  |
|  |  | *g.9430C>A* | *WT* | **43C>A* | *WT* | 3’ UTR variant | *WT* | rs41266429 |  | Benign |  |
|  |  | *g.9440G>A* | *g.9440G>A* | *c.*53G>A* | *c.*53G>A* | 3’ UTR variant | 3’ UTR variant | rs476220 | rs476220 | Benign | Benign |
| **13** | **#610** | *g.9372C>T* | *WT* | *c.798C>T* | *WT* | p.Asn266Asn | WT | rs35983826 |  | Benign |  |
|  |  | *g.9440G>A* | *g.9440G>A* | *c.*53G>A* | *c.*53G>A* | 3’ UTR variant | 3’ UTR variant | rs476220 | rs476220 | Benign | Benign |
| **14** | **#616** | *g.9372C>T* | *WT* | *c.798C>T* | *WT* | p.Asn266Asn | WT | rs35983826 |  | Benign |  |
|  |  | *g.9440G>A* | *WT* | *c.*53G>A* | *WT* | 3’ UTR variant | *WT* | rs476220 |  | Benign |  |
| **15** | **#622** | *g.8931C>T* | *WT* | *c.357C>T* | *WT* | p.Asn119Asn | WT | rs41310442 |  | Benign |  |
| **16** | **#632** | *g.9430C>A* | *WT* | **43C>A* | *WT* | 3’ UTR variant | *WT* | rs41266429 |  | Benign |  |
|  |  | *g.9440G>A* | *WT* | *c.*53G>A* | *WT* | 3’ UTR variant | *WT* | rs476220 |  | Benign |  |
| **17** | **#653** | *g.9372C>T* | *WT* | *c.798C>T* | *WT* | p.Asn266Asn | WT | rs35983826 |  | Benign |  |
|  |  | *g.9440G>A* | *WT* | *c.*53G>A* | *WT* | 3’ UTR variant | *WT* | rs476220 |  | Benign |  |
| **18** | **#654** | *WT* | *WT* | *WT* | *WT* | WT | WT |  |  |  |  |
| **19** | **#657** | *WT* | *WT* | *WT* | *WT* | WT | WT |  |  |  |  |
| **20** | **#659** | *g.9372C>T* | *WT* | *c.798C>T* | *WT* | p.Asn266Asn | WT | rs35983826 |  | Benign |  |
|  |  | *g.9440G>A* | *WT* | *c.*53G>A* | *WT* | 3’ UTR variant | *WT* | rs476220 |  | Benign |  |
| **21** | **#660** | *g.8931C>T* | *WT* | *c.357C>T* | *WT* | p.Asn119Asn | WT | rs41310442 |  | Benign |  |
|  |  | *g.9440G>A* | *WT* | *c.*53G>A* | *WT* | 3’ UTR variant | *WT* | rs476220 |  | Benign |  |
| **22** | **#663** | *g.9430C>A* | *WT* | **43C>A* | *WT* | 3’ UTR variant | *WT* | rs41266429 |  | Benign |  |
|  |  | *g.9440G>A* | *WT* | *c.*53G>A* | *WT* | 3’ UTR variant | *WT* | rs476220 |  | Benign |  |
| **23** | **#666** | *g.9372C>T* | *WT* | *c.798C>T* | *WT* | p.Asn266Asn | WT | rs35983826 |  | Benign |  |
|  |  | *g.9440G>A* | *g.9440G>A* | *c.*53G>A* | *c.*53G>A* | 3’ UTR variant | 3’ UTR variant | rs476220 | rs476220 | Benign | Benign |
| **24** | **#667** | *WT* | *WT* | *WT* | *WT* | WT | WT |  |  |  |  |
| **25** | **#669** | *WT* | *WT* | *WT* | *WT* | WT | WT |  |  |  |  |
| **26** | **#670** | *g.9372C>T* | *WT* | *c.798C>T* | *WT* | p.Asn266Asn | WT | rs35983826 |  | Benign |  |
|  |  | *g.9440G>A* | *WT* | *c.*53G>A* | *WT* | 3’ UTR variant | *WT* | rs476220 |  | Benign |  |
| **27** | **#671** | *WT* | *WT* | *WT* | *WT* | WT | WT |  |  |  |  |
| **28** | **#672** | *g.9440G>A* | *WT* | *c.*53G>A* | *WT* | 3’ UTR variant | *WT* | rs476220 |  | Benign |  |
| **29** | **#678** | *WT* | *WT* | *WT* | *WT* | WT | WT |  |  |  |  |
| **30** | **#679** | *WT* | *WT* | *WT* | *WT* | WT | WT |  |  |  |  |
| **31** | **#680** | *WT* | *WT* | *WT* | *WT* | WT | WT |  |  |  |  |
| **32** | **#681** | *WT* | *WT* | *WT* | *WT* | WT | WT |  |  |  |  |

**Table S10: Sanger sequencing results of *POU3F4.*** NCBI GeneBank Reference Sequences: NG_009936.2, NM_000307.1, NP_00298.3. The clinical significance is given according to Clin Var (<https://www.ncbi.nlm.nih.gov/clinvar/>). N.A., not assessed. * denotes a STOP codon.

|  | **Patient ID** | **Sex** | ***POU3F4***  ***gDNA*** | | ***POU3F4***  ***cDNA*** | | **POU3F4**  **protein** | | **SNP ID** | | **Clinical significance** | |
| --- | --- | --- | --- | --- | --- | --- | --- | --- | --- | --- | --- | --- |
|  |  |  | ***Allele 1*** | ***Allele 2*** | ***Allele 1*** | ***Allele 2*** | **Protein 1** | **Protein 2** | ***Allele 1*** | ***Allele 2*** | ***Allele 1*** | ***Allele 2*** |
| **1** | **#119** | M | *WT* | *-* | *WT* | *-* | WT | - |  |  |  |  |
| **2** | **#271** | F | *WT* | *WT* | *WT* | *WT* | WT | WT |  |  |  |  |
| **3** | **#272** | M | *WT* | *-* | *WT* | *-* | WT | - |  |  |  |  |
| **4** | **#278** | F | *WT* | *WT* | *WT* | *WT* | WT | WT |  |  |  |  |
| **5** | **#305** | M | *WT* | - | *WT* | - | WT | - |  |  |  |  |
| **6** | **#307** | M | *WT* | - | *WT* | - | WT | - |  |  |  |  |
| **7** | **#358** | F | *WT* | *WT* | *WT* | *WT* | WT | WT |  |  |  |  |
| **8** | **#359** | F | *WT* | *WT* | *WT* | *WT* | WT | WT |  |  |  |  |
| **9** | **#395** | F | *WT* | *WT* | *WT* | *WT* | WT | WT |  |  |  |  |
| **10** | **#421** | M | *WT* | *-* | *WT* | *-* | WT | - |  |  |  |  |
| **11** | **#568** | F | *WT* | *WT* | *WT* | *WT* | WT | WT |  |  |  |  |
| **12** | **#569** | M | *g.5284delA* | *-* | *c.220delA* | *-* | p.Ser74Alafs*8 | - | N.A. | - | Unknown | - |
| **13** | **#610** | F | *WT* | *WT* | *WT* | *WT* | WT | WT |  |  |  |  |
| **14** | **#616** | F | *WT* | *WT* | *WT* | *WT* | WT | WT |  |  |  |  |
| **15** | **#622** | M | *WT* | *-* | *WT* | *-* | WT | - |  |  |  |  |
| **16** | **#632** | F | *WT* | *WT* | *WT* | *WT* | WT | WT |  |  |  |  |
| **17** | **#653** | M | *WT* | *-* | *WT* | *-* | WT | - |  |  |  |  |
| **18** | **#654** | F | *WT* | *WT* | *WT* | *WT* | WT | WT |  |  |  |  |
| **19** | **#657** | F | *WT* | *WT* | *WT* | *WT* | WT | WT |  |  |  |  |
| **20** | **#659** | M | *WT* | - | *WT* | - | *WT* | - |  |  |  |  |
| **21** | **#660** | M | *WT* | - | *WT* | - | *WT* | - |  |  |  |  |
| **22** | **#663** | M | *WT* | - | *WT* | - | *WT* | - |  |  |  |  |
| **23** | **#666** | F | *WT* | *WT* | *WT* | *WT* | *WT* | *WT* |  |  |  |  |
| **24** | **#667** | M | *g.6045T>A* | *-* | c.979T>A | - | p.Cys327* | - | N.A. | - | Unknown | - |
| **25** | **#669** | M | *WT* | *WT* | *WT* | *WT* | WT | WT |  |  |  |  |
| **26** | **#670** | F | *WT* | *WT* | *WT* | *WT* | *WT* | *WT* |  |  |  |  |
| **27** | **#671** | F | *WT* | *WT* | *WT* | *WT* | *WT* | *WT* |  |  |  |  |
| **28** | **#672** | M | *WT* | - | *WT* | - | *WT* | - |  |  |  |  |
| **29** | **#678** | F | *WT* | *WT* | *WT* | *WT* | *WT* | *WT* |  |  |  |  |
| **30** | **#679** | F | *WT* | *WT* | *WT* | *WT* | *WT* | *WT* |  |  |  |  |
| **31** | **#680** | M | *WT* | *-* | *WT* | *-* | *WT* | *-* |  |  |  |  |
| **32** | **#681** | M | *WT* | *-* | *WT* | *-* | *WT* | *-* |  |  |  |  |

**Figure S1: Pedigree of the family of index patient #616 (arrow) concerning the CEVA haplotype SNPs #4 (rs79579403) and #10 (rs9649298)** **and SLC26A4 (pendrin) variant p.Y115D (p.Tyr115Asp) and possible genetic configurations of the family members.** Both parents carry the heterozygous CEVA haplotype, while the heterozygous *SLC26A4* variant p.Y115D was found in the mother and the index patient. Both parents have normal hearing. This pedigree can theoretically be explained by genetic configurations 1-3, where both alleles are represented for each individual. In genetic configuration 1, the index patient inherited both the CEVA haplotype and the *SLC26A4* variant from her mother. This configuration is non-diagnostic, as the CEVA and the pendrin variant are *in cis* in the index patient, and the second causative allele would be missing. It is also unlikely for the unaffected mother to have the same genetic configuration as the affected index patient unless the mother has EVA but normal hearing, which could not be determined. In genetic configuration 2, the index patient inherited the heterozygous CEVA haplotype from her father and the *SLC26A4* variant from her mother. Again, the unaffected mother would have the same genotype as the affected index patient, which is unlikely. In configuration 3, a crossing-over event (red dotted line) would permit the CEVA haplotype to be *in trans* with the pathogenic *SLC26A4* variant, thus explaining the index patient´s phenotype.

**Figure S2. Pedigree of the family of index patient #671 (arrow) concerning the CEVA haplotype SNP #10 (rs9649298)** **and *SLC26A4* (pendrin) variant c.1001+1G>A and possible genetic configurations of the family members.** Only the normal-hearing brother was available for gene analysis. The index patient and her brother carry the heterozygous CEVA haplotype and the heterozygous c.1001+1G>A *SLC26A4* variant, while the genotype of the parents is unknown (?). This pedigree can be theoretically explained by genetic configurations 1-3, where both alleles are represented for each individual and the possible genotype of parents is shown. In genetic configuration 1, the index patient and her brother inherited both the CEVA haplotype and the *SLC26A4* variant from the same parent. Genetic configuration 1 is non-diagnostic, as the CEVA and the *SLC26A4* variant are *in cis* in the index patient, and the second causative allele would be missing. It is also unlikely for the unaffected brother to have the same genotype as the affected index patient unless the brother has EVA but normal hearing, which could not be determined. In genetic configuration 2, the index patient inherited the CEVA haplotype from one parent and the *SLC26A4* variant from the other parent. Again, the unaffected brother would have the same genotype as the affected index patient, which is unlikely. In configuration 3, a crossing-over event only occurring in index patient #671 (red dotted line) would permit the CEVA haplotype to be *in trans* with the pathogenic *SLC26A4* c.1001+1G>A variant, thus explaining the index patient´s phenotype and normal hearing in the brother. In this family, there is also a sister with congenital hearing loss (Table S3) who was unavailable for gene analysis.

**Figure S3. Pedigree of the family of index patient #653 (arrow) concerning the CEVA haplotype SNPs #4 (rs79579403) and #10 (rs9649298)** **and SLC26A4 (pendrin) variant p.G740V (p.Gly740Val) and genetic configuration of the family members.** The mother of the index patient carried a partial (9 of the 12 SNPs) heterozygous CEVA haplotype, while the father carried the complete heterozygous CEVA haplotype and the benign SLC26A4 variant p.G740V. Both parents have normal hearing. The index patient inherited the partial CEVA haplotype from his mother and the complete CEVA haplotype from his father, where the CEVA haplotype co-segregated with the benign SLC26A4 variant p.G740V (grey circle). This would imply that the partial CEVA haplotype is causative when found *in trans* with the complete CEVA haplotype.

**Figure S4. Pedigree of the family of index patient #659 (arrow) concerning the CEVA haplotype SNP #10 (rs9649298)** **and SLC26A4 (pendrin) variants p.A664V (p.Ala664Val) and p.I136N (p.Ile136Asn) and genetic configuration of the family members.** The mother of the index patient carried the heterozygous CEVA haplotype and the pathogenic SLC26A4 variant p.I136N, while the father carried the pathogenic SLC26A4 variant p.A664V. Both parents have normal hearing. The index patient inherited one *SLC26A4* variant from his father and another *SLC26A4* variant and the CEVA haplotype from his mother. Also in this family, the CEVA haplotype appears to segregate with a *SLC26A4* variant.

**References**

1. DiCiommo DP, Duckett A, Burcescu I, Bremner R, Gallie BL. (2004) Retinoblastoma protein purification and transduction of retina and retinoblastoma cells using improved alphavirus vectors. *Invest Ophthalmol Vis Sci* **45:** 3320-3329.

2. Adler J, Parmryd I. (2010) Quantifying colocalization by correlation: the Pearson correlation coefficient is superior to the Mander's overlap coefficient. *Cytometry. Part A.* **77:** 733-742.

3. Roesch S*, et al.* (2018) Functional Testing of SLC26A4 Variants-Clinical and Molecular Analysis of a Cohort with Enlarged Vestibular Aqueduct from Austria. *Int J Mol Sci.* **19:** 209.
